# High-coverage plasma lipidomics reveals novel sex-specific lipidomic fingerprints of age and BMI: Evidence from two large population cohort studies

**DOI:** 10.1371/journal.pbio.3000870

**Published:** 2020-09-28

**Authors:** Habtamu B. Beyene, Gavriel Olshansky, Adam Alexander T. Smith, Corey Giles, Kevin Huynh, Michelle Cinel, Natalie A. Mellett, Gemma Cadby, Joseph Hung, Jennie Hui, John Beilby, Gerald F. Watts, Jonathan S. Shaw, Eric K. Moses, Dianna J. Magliano, Peter J. Meikle

**Affiliations:** 1 Baker Heart and Diabetes Institute, Melbourne, Australia; 2 Faculty of Medicine, Nursing and Health Sciences, Monash University, Melbourne, Australia; 3 School of Population and Global Health, University of Western Australia, Perth, Australia; 4 Medical School, Faculty of Health and Medical Sciences, University of Western Australia, Perth, Australia; 5 PathWest Laboratory Medicine of Western Australia, Nedlands, Western Australia; 6 Lipid Disorders Clinic, Department of Cardiology, Royal Perth Hospital, Perth, Australia; 7 Menzies Institute for Medical Research, University of Tasmania, Tasmania, Australia; 8 School of Public Health and Preventive Medicine, Monash University, Melbourne, Australia; Duke University, UNITED STATES

## Abstract

Obesity and related metabolic diseases show clear sex-related differences. The growing burden of these diseases calls for better understanding of the age- and sex-related metabolic consequences. High-throughput lipidomic analyses of population-based cohorts offer an opportunity to identify disease-risk–associated biomarkers and to improve our understanding of lipid metabolism and biology at a population level. Here, we comprehensively examined the relationship between lipid classes/subclasses and molecular species with age, sex, and body mass index (BMI). Furthermore, we evaluated sex specificity in the association of the plasma lipidome with age and BMI. Some 747 targeted lipid measures, representing 706 molecular lipid species across 36 classes/subclasses, were measured using a high-performance liquid chromatography coupled mass spectrometer on a total of 10,339 participants from the Australian Diabetes, Obesity and Lifestyle Study (AusDiab), with 563 lipid species being validated externally on 4,207 participants of the Busselton Health Study (BHS). Heat maps were constructed to visualise the relative differences in lipidomic profile between men and women. Multivariable linear regression analyses, including sex-interaction terms, were performed to assess the associations of lipid species with cardiometabolic phenotypes. Associations with age and sex were found for 472 (66.9%) and 583 (82.6%) lipid species, respectively. We further demonstrated that age-associated lipidomic fingerprints differed by sex. Specific classes of ether-phospholipids and lysophospholipids (calculated as the sum composition of the species within the class) were inversely associated with age in men only. In analyses with women alone, higher triacylglycerol and lower lysoalkylphosphatidylcholine species were observed among postmenopausal women compared with premenopausal women. We also identified sex-specific associations of lipid species with obesity. Lysophospholipids were negatively associated with BMI in both sexes (with a larger effect size in men), whilst acylcarnitine species showed opposing associations based on sex (positive association in women and negative association in men). Finally, by utilising specific lipid ratios as a proxy for enzymatic activity, we identified stearoyl CoA desaturase (SCD-1), fatty acid desaturase 3 (FADS3), and plasmanylethanolamine Δ1-desaturase activities, as well as the sphingolipid metabolic pathway, as constituent perturbations of cardiometabolic phenotypes. Our analyses elucidate the effect of age and sex on lipid metabolism by offering a comprehensive view of the lipidomic profiles associated with common cardiometabolic risk factors. These findings have implications for age- and sex-dependent lipid metabolism in health and disease and suggest the need for sex stratification during lipid biomarker discovery, establishing biological reference intervals for assessment of disease risk.

## Abstract 2

Obesity and related metabolic diseases show clear age- and sex-related differences. A high-throughput targeted lipidomics study of two independent human cohorts defines detailed molecular fingerprints of age, sex, and body mass index, identifying novel interactions between these metabolic risk factors.

## Introduction

Cardiometabolic conditions including obesity, type 2 diabetes (T2D), and cardiovascular disease (CVD) are tightly associated with dysregulation of lipid metabolism [1–3], which contributes directly or indirectly to adverse metabolic outcomes. Elevated total cholesterol, triglycerides, and low-density lipoprotein cholesterol (LDL-C) and decreased high-density lipoprotein cholesterol (HDL-C) are used as measures of metabolic health status and disease risk [4–6]. However, such clinical lipid measures do not adequately explain the complex pathophysiology of metabolic disease, nor do they suffice as diagnostic, prognostic, or predictive biomarkers. Defining the relationship between cardiometabolic risk factors and individual lipid species helps to identify potential biomarkers associated with disease risk and to understand the metabolic basis and pathophysiology of cardiometabolic diseases.

The human plasma lipidome is composed of many hundreds to thousands of molecular lipid species, displaying an enormous structural and functional diversity [7–9]. Yet, there is limited understanding about the relationship of these molecular components with cardiometabolic risk. Many lipid species have been shown to be altered during the onset and progression of cardiometabolic diseases such as T2D and CVD [10–13]. However, whilst evidence of the association of lipid species with cardiometabolic risk factors or disease outcomes on small cohorts has been well documented [10, 13, 14], there have been relatively few studies involving large population-based cohorts. In a cohort of 1,000 participants, Weir and colleagues utilised liquid chromatography tandem mass-spectrometry (LC-MS/MS)–based targeted lipidomics and profiled major lipid classes (23) and species (312) in human plasma, identifying lipid classes and subclasses associated with cardiometabolic risk factors [15]. More recently, Huynh and colleagues reported detailed associations of lipid species with anthropometric and insulin resistance measures in a subcohort (*n* = 640) selected from the Australian Diabetes, Obesity and Lifestyle Study (AusDiab) [16]. In addition to the limitations of small sample numbers, most previous studies have used targeted mass-spectrometry–based lipidomics with a limited number of lipid species [14, 16–19]. Advances in high-throughput LC-MS/MS–based lipidomic profiling now allow the measurement of many hundreds of biologically relevant circulating molecular lipid species, providing a more complete picture of the lipidome [16, 20].

The interaction of age and sex with metabolism in disease settings is well recognised [21–23]. Of note, the risk at time of onset and pathology of CVD has been shown to vary depending on sex [24]. Cholesterol metabolism is regulated in a sex-specific manner and is associated with distinct risk profiles in men and women [25]. Several investigators have shown that metabolite fingerprints in men and women vary significantly in an age-specific manner [26, 27]. Understanding the age and sex interaction within lipid metabolism is important for biomarker identification and will be necessary for precision medicine. Whilst the levels of nonlipid metabolites and a few lipid or lipoprotein classes have been shown to differ in age- and sex-specific fashions, evidence on age- and sex-specific lipidomic fingerprints, as well as the effect of menopause, is lacking. The identification of sex-specific associations of lipid species with cardiometabolic risk factors is essential to understanding crosstalk between lipid metabolism and sex that may underlie differential risk, and to obtain a holistic view of lipid metabolism in these disease processes.

Using a targeted LC-MS/MS method, we measured 706 individual lipid species across 36 distinct lipid classes from the blood of 10,339 adult Australians, aged between 25 to 95 years, from the AusDiab study. We examined the associations between the plasma lipidome and common cardiometabolic risk factors including age, sex, and body mass index (BMI). We further tested whether the relationship between age or BMI and lipidomic profile differs by sex to gain biological insight into the potential mechanism of lipid dysregulation during obesity in men and women. We validated our results measuring 563 lipid species in 4,207 participants of the Busselton Health Study (BHS).

## Results

### The plasma lipidome is sex-specific

The variability in the levels of plasma lipid species between men and women was generally similar (% coefficient of variation [CV] = 14–170). However, the coefficient of variation for many diacylglycerol, triacylglycerol, and alkyl-diacylglycerol species tends to be higher in men (S1 Fig). The correlation structure of the lipidome showed strong correlations between species both within and between classes (S2 Fig, S1 Table). We could also observe a negative correlation structure, particularly for di- and triacylglycerol species against phospholipid and sphingolipid species. The correlation structure in men (S3 Fig, S2 Table) and women (S4 Fig, S3 Table) appeared similar. However, subtraction of one from the other identified many differences (S5 Fig, S4 Table).

**Fig 1:**
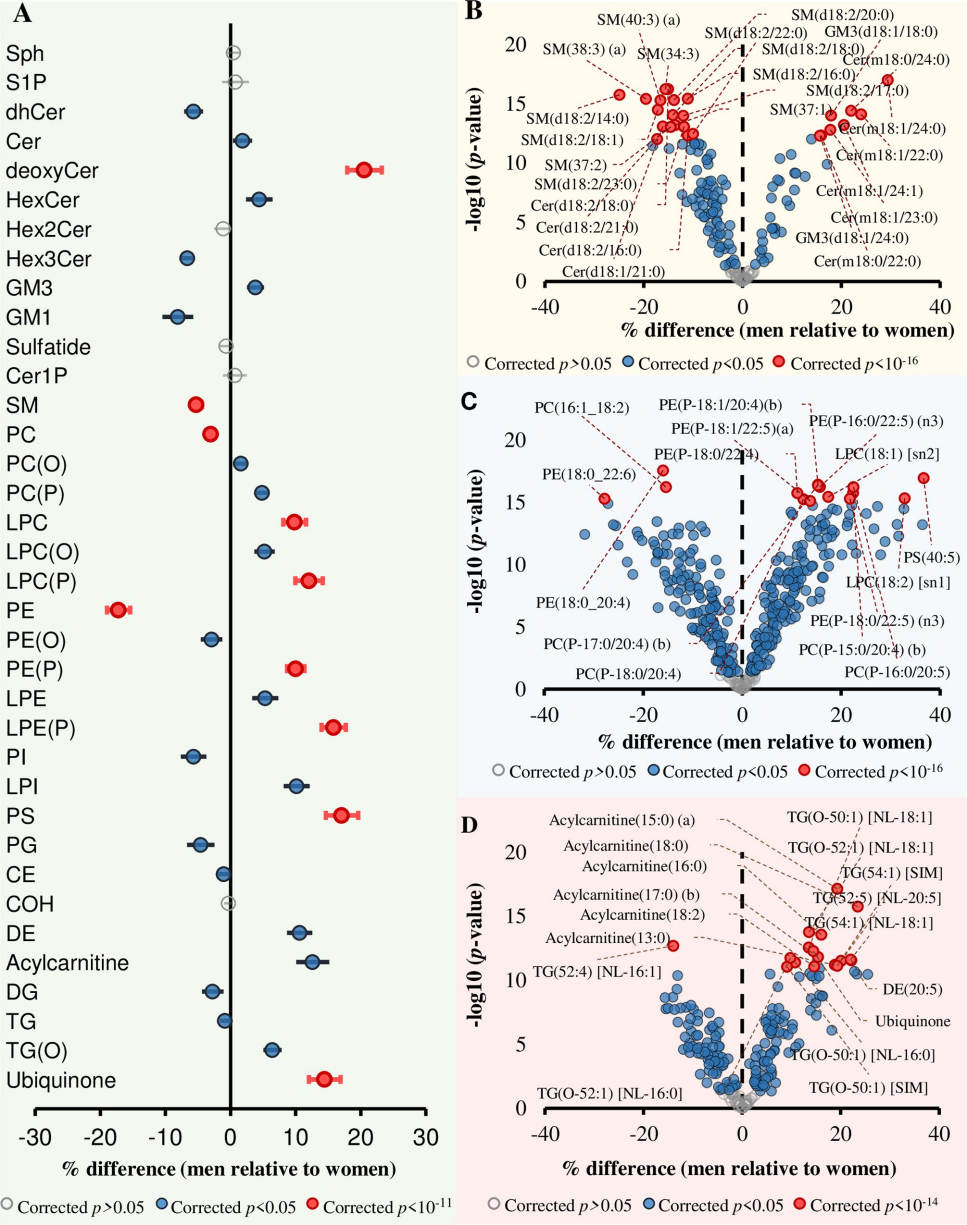
Associations between sex and lipid classes, subclasses, and species. Linear regression analysis between sex and log-transformed concentrations of each lipid species was performed adjusting for age, BMI, total cholesterol, HDL-C, and triglycerides (*n* = 10,339). The beta coefficients were converted to percentage difference for men relative to women. (A) Associations between sex and lipid classes/subclasses. (B) Associations between sex and sphingolipid species. (C) Associations between sex and phospholipid species. (D) Associations between sex and glycerolipid and fatty-acyl species. Grey circles show nonsignificant classes/subclasses and species, blue circles show classes/subclasses and species with corrected *p* < 0.05, and pink circles represent the most significantly associated classes/subclasses and species. Whiskers represent 95% confidence intervals. The underlying data can be found in S1 Data. BMI, body mass index; CE, cholesteryl ester; Cer, ceramide; Cer-1-P, ceramide-1-phosphate; COH, free cholesterol; DE, dehydrocholesterol; deoxyCer, deoxyceramide; DG, diacylglycerol; dhCer, dihydroceramide; GM1, G_M1_ ganglioside; GM3, G_M3_ ganglioside; HDL-C, high-density lipoprotein cholesterol; HexCer, monohexosylceramide; Hex2Cer, dihexosylceramide; Hex3Cer, trihexosylceramide; LPC, lysophosphatidylcholine; LPC(O), lysoalkylphosphatidylcholine; LPC(P), lysoalkenylphosphatidylcholine; LPE, lysophosphatidylethanolamine; LPE(P), lysoalkenylphosphatidylethanolamine; LPI, lysophosphatidylinositol; NL, neutral loss; PC, phosphatidylcholine; PC(O), alkylphosphatidylcholine; PC(P), alkenylphosphatidylcholine; PE, phosphatidylethanolamine; PE(O), alkylphosphatidylethanolamine; PE(P), alkenylphosphatidylethanolamine; PG, phosphatidylglycerol; PI, phosphatidylinositol; SM, sphingomyelin; sn, stereospecifically numbered; Sph, sphingosine; S-1-P, sphingosine-1-phosphate; TG, triacylglycerol; TG(O), alkyl-diacylglycerol.

At the lipid class level, 30 out of 36 classes/subclasses were significantly associated with sex after adjusting for age, BMI, total cholesterol, HDL-C, and triglycerides (corrected *p* < 0.05). “Corrected *p*” throughout this paper refers to false discovery rate (FDR) correction using the Benjamini–Hochberg procedure. Based on *p*-values, lysoalkenylphosphatidylethanolamine displayed the strongest difference between sexes (15.8% higher in men relative to women, corrected *p*-value = 1.25 × 10^−15^) (Fig 1A).

Class-level analyses do not capture the detailed differences in the fatty-acyl chain length and double-bond content that characterise individual lipid species. Therefore, we extended our analysis in relation to molecular lipid species. A total of 583 (82.6%) lipid species were associated with sex (*p* < 0.05) after correction for multiple comparisons. Within the sphingolipid classes, being male was strongly associated with lower levels of sphingomyelin and higher deoxyceramide species (Fig 1B). Within the sphingomyelin class, SM(18:2/14:0) displayed the most significant difference between sexes (24.9% lower in men relative to women, *p* = 1.79 × 10^−16^). Deoxyceramide species were higher in men, with Cer(m18:1/24:0) showing the strongest association with sex (29.4% higher in men, *p* = 9.87 × 10^−18^). Ceramide species were generally lower in men except for those containing a 24:0 fatty-acyl chain, which showed a significant positive association with men (Fig 1B). From the glycerophospholipid category, lower phosphatidylcholine and phosphatidylethanolamine and higher lyso- and ether-phospholipid levels were associated with being male (Fig 1C). The majority of acylcarnitine, triacylglycerol, and alkyl-diacylglycerol species were significantly higher in men compared with women (Fig 1D).

**Fig 2:**
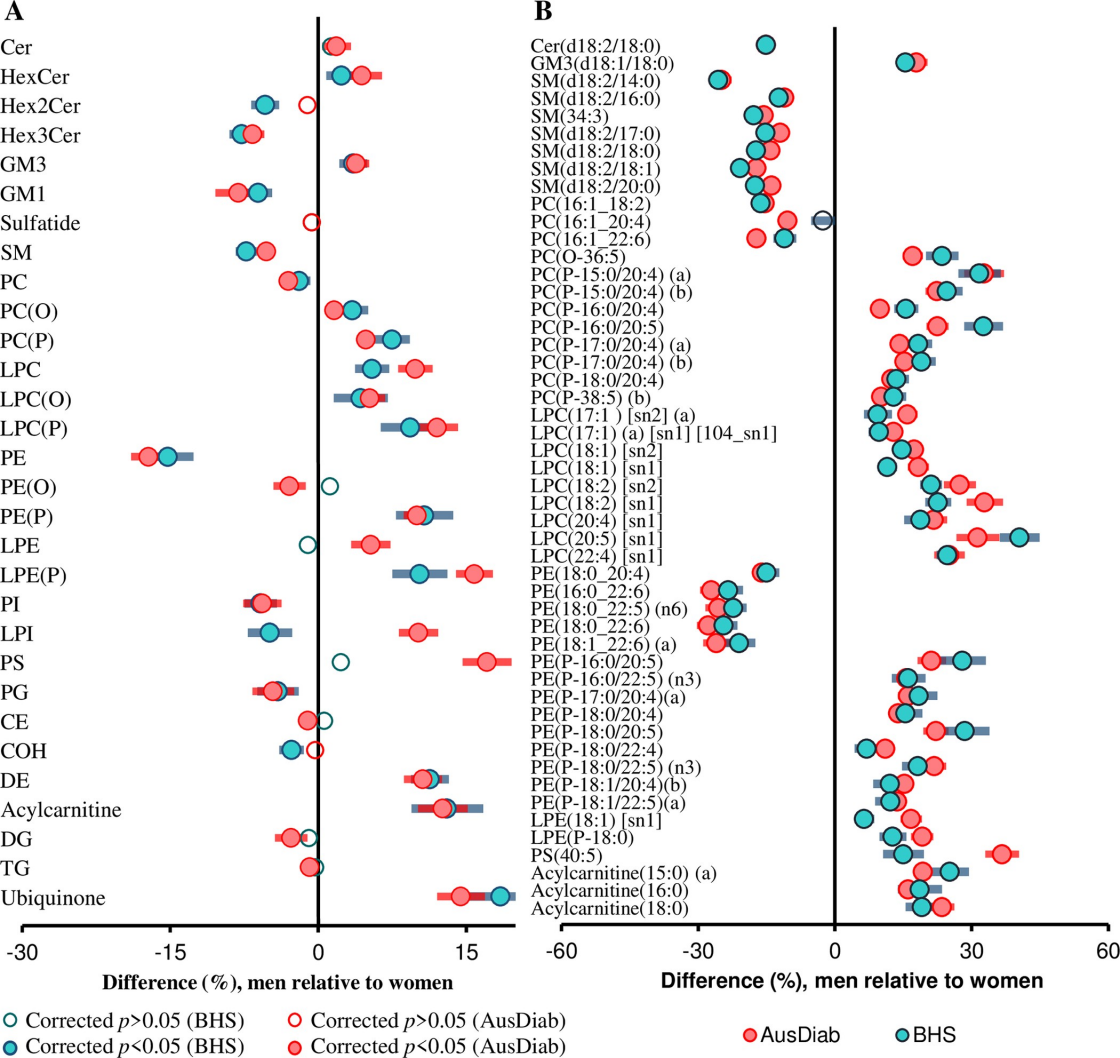
Validation of the associations between sex and plasma lipid classes and species. Linear regression analysis between sex and log-transformed lipid concentrations was performed adjusting for age, BMI, total cholesterol, HDL-C, and triglycerides on 10, 339 subjects in the AusDiab cohort and 4,207 in Busselton at a class level (A) and the 50 most significant lipid species (B). Blue (men) and pink (women) open circles show lipid classes/subclasses or species with corrected *p* > 0.05. Closed circles show classes/subclasses or species with corrected *p* < 0.05. Whiskers represent 95% confidence intervals. See S1 Data for the underlying data. AusDiab, Australian Diabetes, Obesity and Lifestyle Study; BMI, body mass index; CE, cholesteryl ester; Cer, ceramide; COH, free cholesterol; DE, dehydrocholesterol; DG, diacylglycerol; GM1, G_M1_ ganglioside; GM3, G_M3_ ganglioside; HDL-C, high-density lipoprotein cholesterol; HexCer, monohexosylceramide; Hex2Cer, dihexosylceramide; Hex3Cer, trihexosylceramide; LPC, lysophosphatidylcholine; LPC(O), lysoalkylphosphatidylcholine; LPC(P), lysoalkenylphosphatidylcholine; LPE, lysophosphatidylethanolamine; LPE(P), lysoalkenylphosphatidylethanolamine; LPI, lysophosphatidylinositol; PC, phosphatidylcholine; PC(O), alkylphosphatidylcholine; PC(P), alkenylphosphatidylcholine; PE, phosphatidylethanolamine; PE(O), alkylphosphatidylethanolamine; PE(P), alkenylphosphatidylethanolamine; PG, phosphatidylglycerol; PI, phosphatidylinositol; PS, phosphatidylserine; SM, sphingomyelin; TG, triacylglycerol.

To validate our results, we utilised an independent cohort of 4,207 participants from the BHS, in which lipidomic data were available for 30 lipid classes/subclasses and 563 lipid species. For replication, we recalculated the lipid classes/subclasses using only the 563 common lipid species. Nineteen of the 27 classes/subclasses associated with sex in the AusDiab cohort were also significantly associated with sex, with similar effect sizes, in the Busselton cohort (Fig 2A). In addition, of the 563 lipid species common to both cohorts, 478 were associated with sex in the AusDiab cohort, and 408 of these were replicated in Busselton at a corrected *p*-value threshold of 0.05 (S5 Table). Only 8 lipid species showed opposing associations in the 2 cohorts. Of the 50 most significant species associated with sex in the AusDiab cohort (corrected *p* < 7.76 × 10^−14^), 49 were replicated in the Busselton cohort (*p* < 6.60 × 10^−7^). Only one species—PC(16:1_20:4)—was not significant in the Busselton cohort at a corrected *p*-value threshold of 0.05 (Fig 2B). Overall, there was a strong correlation between the regression coefficients for sex in the AusDiab and the Busselton cohorts (r^2^ = 0.840, S6 Fig).

### The association of age with the plasma lipidome

**Fig 3:**
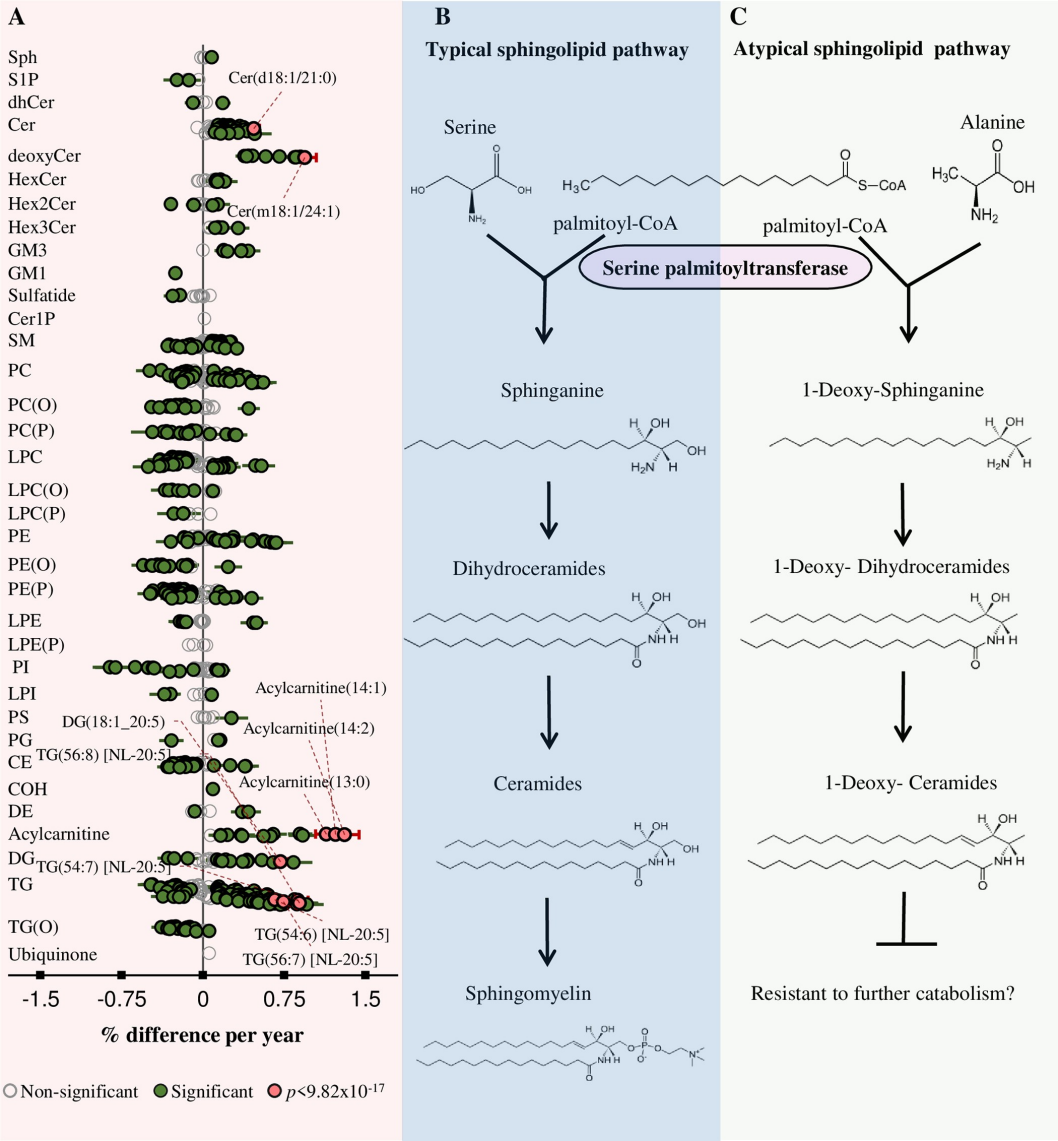
Associations between age and plasma lipid species. (A) Linear regression between age and log-transformed lipid species concentration on 10,339 individuals adjusting for sex, BMI, total cholesterol, HDL-C, and triglyceride levels. Grey circles show nonsignificant species, green show species with *p* < 0.05, and pink show the 10 most significant species after correction for multiple comparisons (*p* < 9.82 × 10^−17^). Whiskers represent 95% confidence intervals. (B) The typical sphingolipid pathway in which SPT joins canonical substrates serine and palmitoyl-CoA to produce the sphinganine base, a precursor for simple and complex sphingolipids. (C) SPT can also utilise alanine instead of serine to produce 1-deoxysphinganine and deoxyCers that lack an OH group at the C1-position. See S1 Data for the underlying data. BMI, body mass index; CE, cholesteryl ester; Cer, ceramide; Cer-1-P, ceramide-1-phosphate; COH, free cholesterol; DE, dehydrocholesterol; deoxyCer, deoxyceramide; DG, diacylglycerol; dhCer, dihydroceramide; GM1, G_M1_ ganglioside; GM3, G_M3_ ganglioside; HDL-C, high-density lipoprotein cholesterol; HexCer, monohexosylceramide; Hex2Cer, dihexosylceramide; Hex3Cer, trihexosylceramide; LPC, lysophosphatidylcholine; LPC(O), lysoalkylphosphatidylcholine; LPC(P), lysoalkenylphosphatidylcholine; LPE, lysophosphatidylethanolamine; LPE(P), lysoalkenylphosphatidylethanolamine; LPI, lysophosphatidylinositol; NL, neutral loss; OH, hydroxyl; PC, phosphatidylcholine; PC(O), alkylphosphatidylcholine; PC(P), alkenylphosphatidylcholine; PE, phosphatidylethanolamine; PE(O), alkylphosphatidylethanolamine; PE(P), alkenylphosphatidylethanolamine; PG, phosphatidylglycerol; PI, phosphatidylinositol; PS, phosphatidylserine; SM, sphingomyelin; Sph, sphingosine; SPT, serine palmitoyltransferase; S-1-P, sphingosine-1-phosphate; TG, triacylglycerol; TG(O), alkyl-diacylglycerol.

In the AusDiab cohort, linear regression of age against lipid species—adjusting for sex, BMI, total cholesterol, HDL-C, and triglycerides—identified a total of 472 plasma lipid species significantly associated with age after correction for multiple comparisons (Fig 3A). Age was strongly associated with ceramide species (Fig 3B) and deoxyceramide species, which arise from an atypical sphingolipid de novo synthesis pathway (Fig 3C). A specific set of triacylglycerol species, containing eicosapentaenoic acid (EPA) (20:5) fatty acids, were positively associated with age, even after adjusting for clinical measures of triglycerides (Fig 3A). Most of the associations seen in the AusDiab cohort were replicated in the Busselton cohort. Meta-analysis of the AusDiab and Busselton cohorts revealed remarkable positive associations of age with acylcarnitine and ceramide species; with acylcarnitine(14:2) displaying the strongest association (% difference per year = 1.368, corrected *p*-value = 1.024x10^−240^). Ether-phospholipids—particularly alkylphosphatidylcholine, alkylphosphatidylethanolamine, and alkenylphosphatidylethanolamine species—were inversely associated with age (S7 Fig).

### Age-related differences of circulating lipidomic profiles are sex-dependent

**Fig 4:**
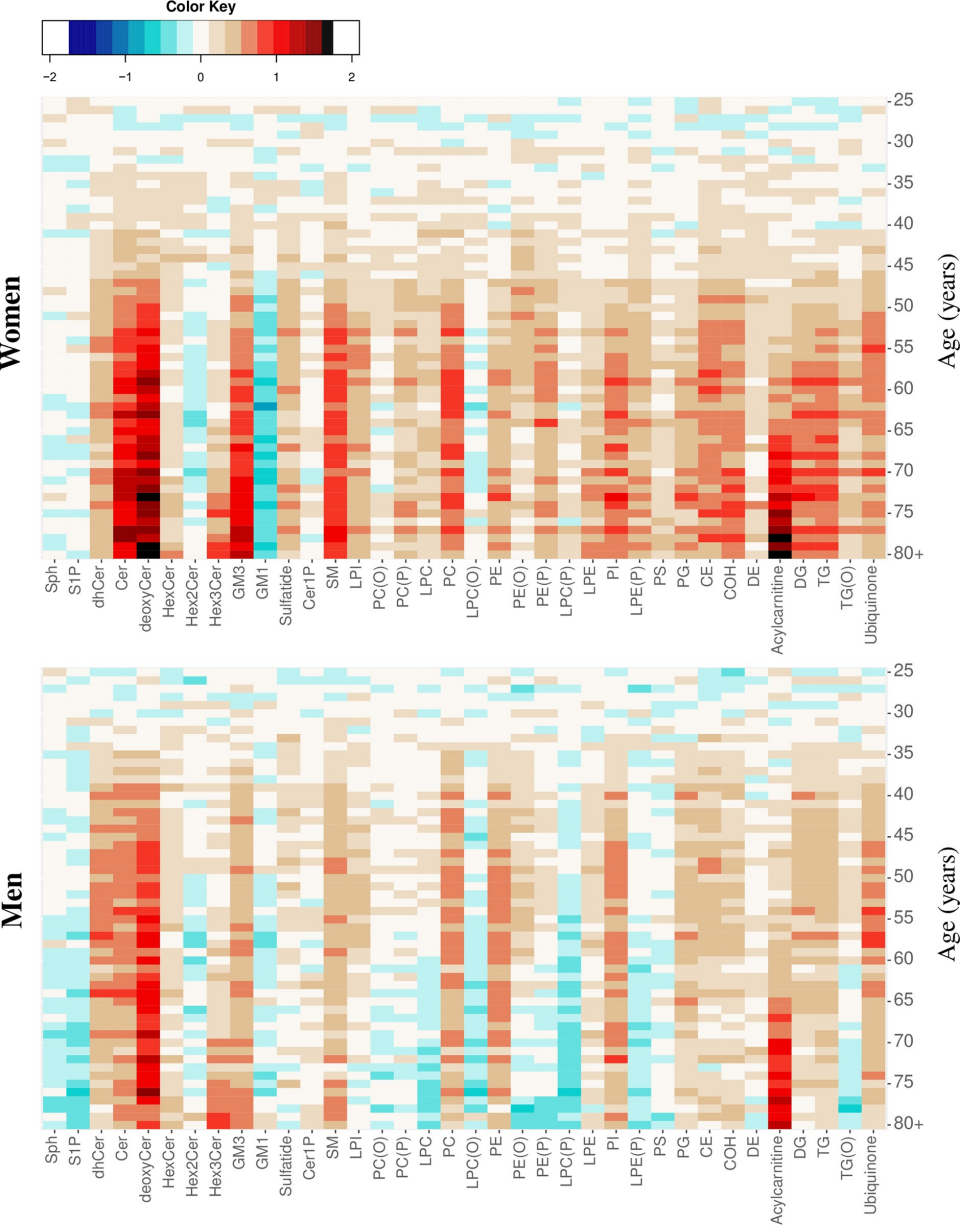
Age- and sex-related differences in plasma lipid classes in the AusDiab cohort. Heat maps showing differences in lipid class/subclass levels across a 1-year age interval constructed separately for men (lower panel) and women (upper panel) after adjusting for BMI. Average lipid class levels were calculated for each 1-year age interval and then centred and scaled to a ‘reference’ group corresponding to 25- to 34-year–old participants. Age groups (by 1-year intervals) are displayed on the *y* axis and the lipid classes on the *x* axis. Colour intensities represent the number of standard deviations away from the mean lipid class levels of the reference group. AusDiab, Australian Diabetes, Obesity and Lifestyle Study; BMI, body mass index; CE, cholesteryl ester; Cer, ceramide; Cer-1-P, ceramide-1-phosphate; COH, free cholesterol; DE, dehydrocholesterol; deoxyCer, deoxyceramide; DG, diacylglycerol; dhCer, dihydroceramide; GM1, G_M1_ ganglioside; GM3, G_M3_ ganglioside; HexCer, monohexosylceramide; Hex2Cer, dihexosylceramide; Hex3Cer, trihexosylceramide; LPC, lysophosphatidylcholine; LPC(O), lysoalkylphosphatidylcholine; LPC(P), lysoalkenylphosphatidylcholine; LPE, lysophosphatidylethanolamine; LPE(P), lysoalkenylphosphatidylethanolamine; LPI, lysophosphatidylinositol; PC, phosphatidylcholine; PC(O), alkylphosphatidylcholine; PC(P), alkenylphosphatidylcholine; PE, phosphatidylethanolamine; PE(O), alkylphosphatidylethanolamine; PE(P), alkenylphosphatidylethanolamine; PG, phosphatidylglycerol; PI, phosphatidylinositol; PS, phosphatidylserine; SM, sphingomyelin; Sph, sphingosine; S-1-P, sphingosine-1-phosphate; TG, triacylglycerol; TG(O), alkyl-diacylglycerol.

To explore age- and sex-related differences in lipidomic profile, we constructed heat maps of lipid classes/subclasses in the AusDiab cohort. The mean lipid level was computed for each 1-year age interval, then normalised to a ‘metabolically healthy’ reference group (25–34 years old) so that the number of standard deviations by which the mean level differed from the reference group was depicted for each lipid. Heat maps were adjusted for BMI by using the residuals of the linear regression of lipids against BMI in the model. The lipidomic profiles across age groups at the lipid class level in women (Fig 4, upper panel) was clearly distinct from men (Fig 4, lower panel), particularly for lyso- and ether-linked phospholipids and for the ether-linked glycerolipid alkyl-diacylglycerol, which tend to decrease with age in men but not in women.

Of those lipid classes that increased with age, the increases in deoxyceramide and G_M3_ ganglioside were stronger in women compared to men. Changes in the lipidomic profile of women appeared to occur at older ages than among men, with changes greater than 0.5 standard deviation units not occurring until 48 years of age in women but at about 38 years in men (Fig 4). Further adjustments for plasma total cholesterol, HDL-C, and triglycerides resulted in fewer lipid classes being different between men and women (S8 Fig). Heat maps for individual lipid species were also constructed, and these also showed differences between men and women (S9 Fig).

**Fig 5:**
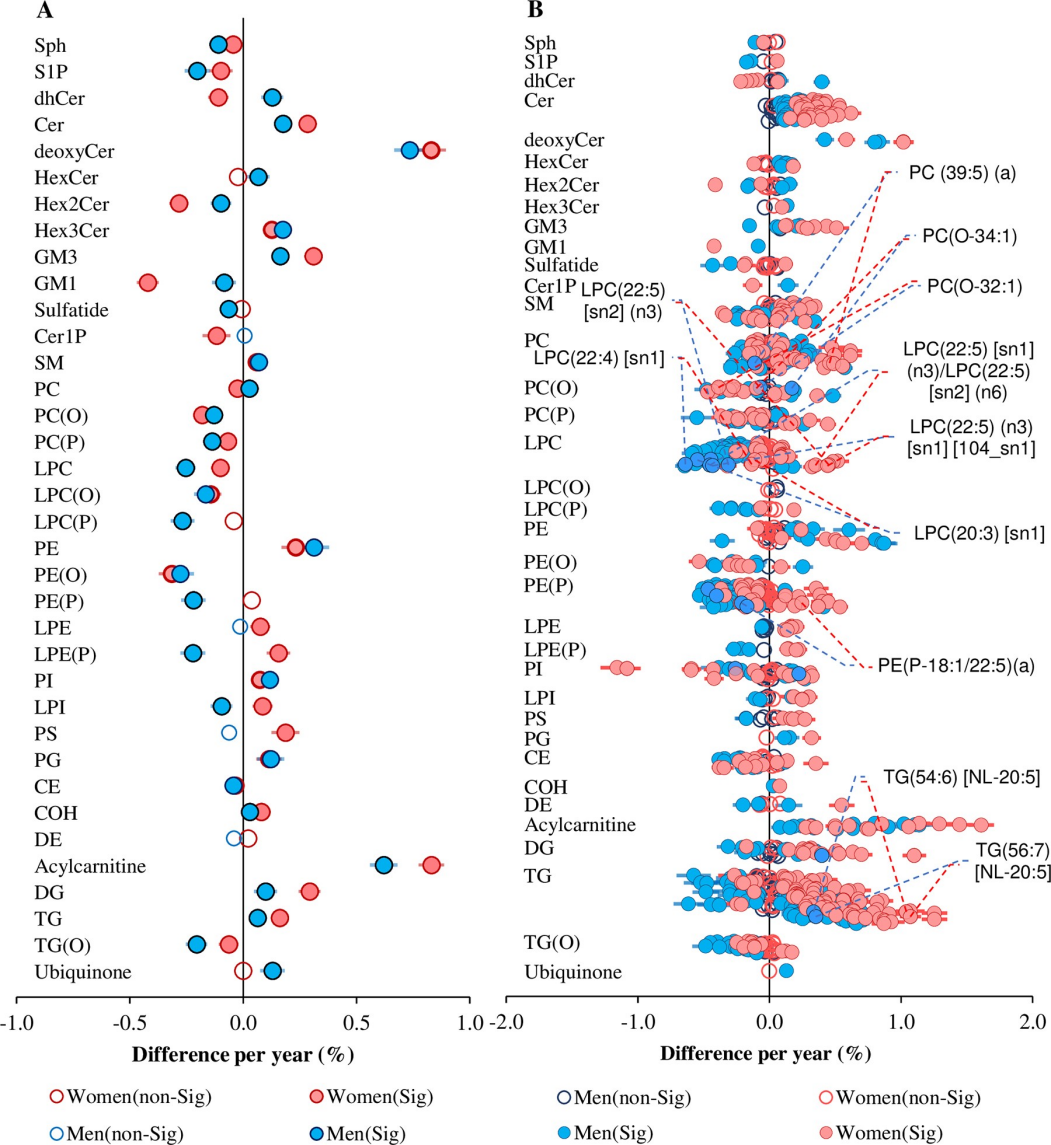
Sex-dependent associations of lipid classes/subclasses and species with age. Linear regression of age against lipid classes/subclasses (A) and lipid species (B) adjusting for sex, BMI, total cholesterol, HDL-C, and triglycerides and including an age × sex interaction term. Pink and blue solid circles show classes and species that are significantly associated with age in women and men respectively (corrected *p* < 0.05). Open circles represent nonsignificant classes/subclasses or species (corrected *p* > 0.05). All lipid classes/subclasses showing significant sex interaction (interaction *p* < 0.05) and the 10 most significantly different species between men and women (interaction *p*-value < 1.0 × 10^−22^) in the association with age are labelled. Whiskers represent 95% confidence intervals. See S1 Data for the underlying data. BMI, body mass index; CE, cholesteryl ester; Cer, ceramide; Cer-1-P, ceramide-1-phosphate; COH, free cholesterol; DE, dehydrocholesterol; deoxyCer, deoxyceramide; DG, diacylglycerol; dhCer, dihydroceramide; GM1, G_M1_ ganglioside; GM3, G_M3_ ganglioside; HDL-C, high-density lipoprotein cholesterol; HexCer, monohexosylceramide; Hex2Cer, dihexosylceramide; Hex3Cer, trihexosylceramide; LPC, lysophosphatidylcholine; LPC(O), lysoalkylphosphatidylcholine; LPC(P), lysoalkenylphosphatidylcholine; LPE, lysophosphatidylethanolamine; LPE(P), lysoalkenylphosphatidylethanolamine; LPI, lysophosphatidylinositol; NL, neutral loss; PC, phosphatidylcholine; PC(O), alkylphosphatidylcholine; PC(P), alkenylphosphatidylcholine; PE, phosphatidylethanolamine; PE(O), alkylphosphatidylethanolamine; PE(P), alkenylphosphatidylethanolamine; PG, phosphatidylglycerol; PI, phosphatidylinositol; PS, phosphatidylserine; SM, sphingomyelin; sn, stereospecifically numbered; Sph, sphingosine; S-1-P, sphingosine-1-phosphate; TG, triacylglycerol; TG(O), alkyl-diacylglycerol.

The heat maps presented both similarities and differences in the lipidomic profiles between men and women across age ranges. We therefore assessed the effect of sex on the association of age with individual lipid species by including an interaction term between sex and age for each lipid species and lipid classes/subclasses adjusting for sex, BMI, total cholesterol, HDL-C, and triglycerides. At the lipid class level, 26 out of the 36 lipid classes/subclasses displayed a significant interaction with sex (Fig 5A). Alkyl- and alkenylphosphatidylcholine and lysophosphatidylcholine tend to be negatively associated with age in both sexes, whilst alkenylphosphatidylethanolamine, lysoalkenylphosphatidylethanolamine, and lysophosphatidylinositol displayed opposing effects (positive in women and negative in men) (Fig 5A).

Several distinct lipid species belonging to the classes mentioned above were found to show opposing effects with age depending on sex. The interaction term was significant for 472 of the 706 lipid species measured (corrected *p*-value < 0.05) (Fig 5B). Species of lysophosphatidylcholine and ether-linked phospholipids showed strong differential association with age based on gender. In particular, omega-3 docosapentaenoic acid (DPA; 22:5) fatty-acid–containing species were highly dependent on age and sex; these species tend to be positively associated with age in women but were negatively associated in men (Fig 5B).

### Association of lipid species with menopause

**Fig 6:**
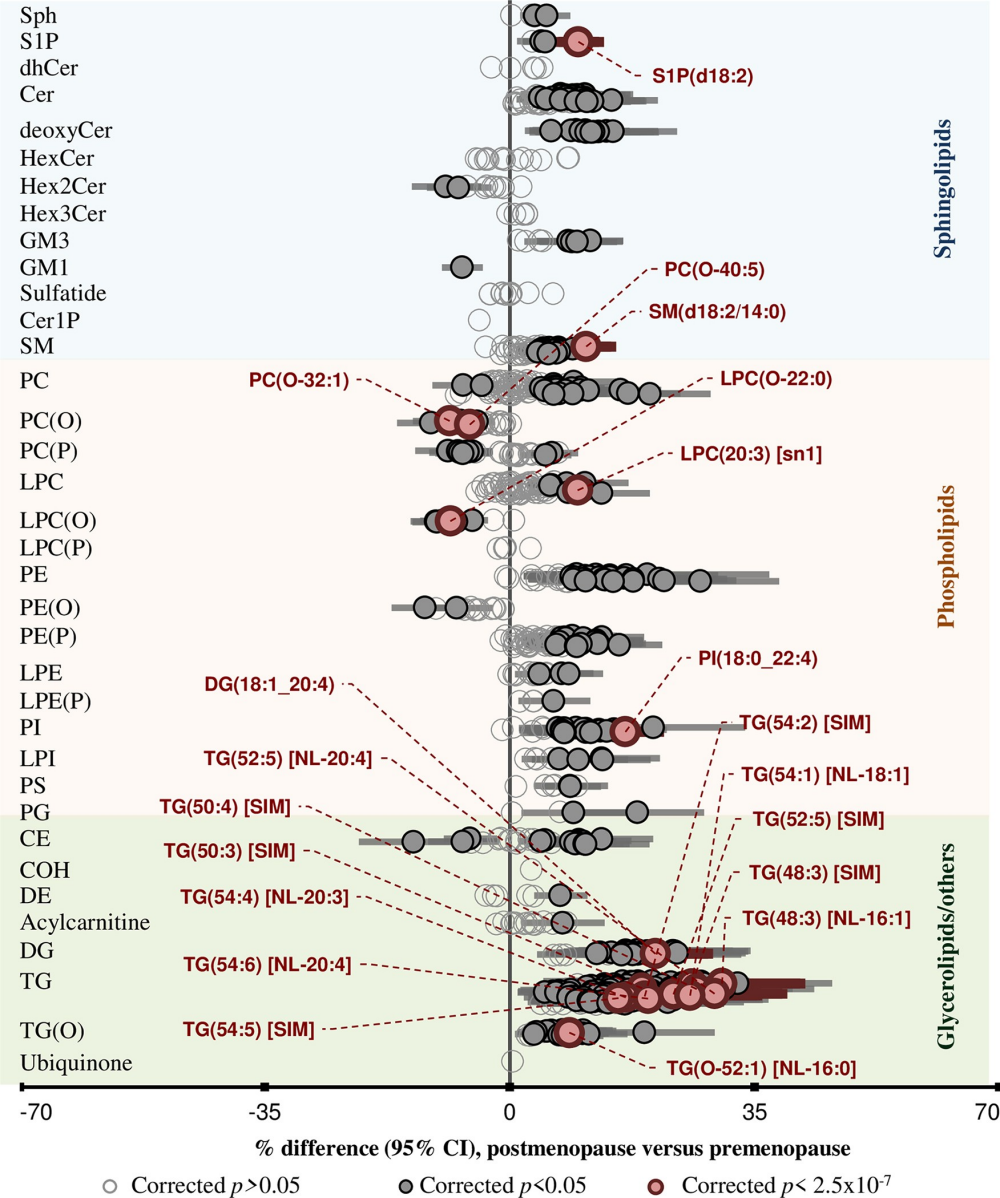
Association of lipid species with menopausal status. Multivariable linear regression analysis between menopausal status (post relative to pre) and log_10_-transformed lipid species concentrations was performed in women only, adjusting for age, BMI, smoking status, and diabetes status. Beta coefficients were converted to percentage difference of lipid level in postmenopausal relative to premenopausal women. Open circles show nonsignificant species (*p* > 0.05). Grey and pink closed circles show species with corrected *p* < 0.05 and corrected *p* < 2.5 × 10^−7^, respectively. See S1 Data for the underlying data. BMI, body mass index; CE, cholesteryl ester; Cer, ceramide; Cer-1-P, ceramide-1-phosphate; COH, free cholesterol; DE, dehydrocholesterol; deoxyCer, deoxyceramide; DG, diacylglycerol; dhCer, dihydroceramide; GM1, G_M1_ ganglioside; GM3, G_M3_ ganglioside; HexCer, monohexosylceramide; Hex2Cer, dihexosylceramide; Hex3Cer, trihexosylceramide; LPC, lysophosphatidylcholine; LPC(O), lysoalkylphosphatidylcholine; LPC(P), lysoalkenylphosphatidylcholine; LPE, lysophosphatidylethanolamine; LPE(P), lysoalkenylphosphatidylethanolamine; LPI, lysophosphatidylinositol; NL, neutral loss; PC, phosphatidylcholine; PC(O), alkylphosphatidycholine; PC(P), alkenylphosphatidylcholine; PE, phosphatidylethanolamine; PE(O), alkylphosphatidylethanolamine; PE(P), alkenylphosphatidylethanolamine; PG, phosphatidylgylcerol; PI, phosphatidylinositol; PS, phosphatidylserine; SIM, single ion monitoring; SM, sphingomyelin; sn, stereospecifically numbered; Sph, sphingosine; S-1-P, sphingosine-1-phosphate; TG, triacylglycerol; TG(O), alkyl-diacylglycerol.

In a subgroup analysis of women, we assessed the association of menopause with the plasma lipidome. Menopausal status was assessed using an interview administered questionnaire. A total of 2,253 reported as postmenopausal and 2,383 as premenopausal; these were included in the analysis (S6 Table). Participants who were not sure about their menopausal status (*n* = 192) or were taking hormone replacement treatment (HRT), such as oestrogen (*n* = 981), were excluded. A multivariable linear regression adjusted for age, BMI, smoking status, and diabetes status was fitted against menopausal status. Lipid species across multiple lipid classes were associated with menopause (Fig 6). These associations were similar to those observed between age and lipid species, despite controlling for age in the analysis. We therefore performed a subanalysis of women in the menopausal transition window (40–60 years old, *n* = 1,920) to define the effect of menopause on the lipidome independent of age. We observed a significant association of menopause with 53 lipid species (Fig 6). Postmenopausal women showed significantly higher phosphatidylinositol, diacylglycerol, triacylglycerol, and alkyl-diacylglycerol levels compared with premenopausal women. Postmenopausal women had lower ether and lysophospholipids, particularly alkylphosphatidylcholine and lysoalkylphosphatidylcholine species (Fig 6).

### The association between BMI and the plasma lipidome

**Fig 7:**
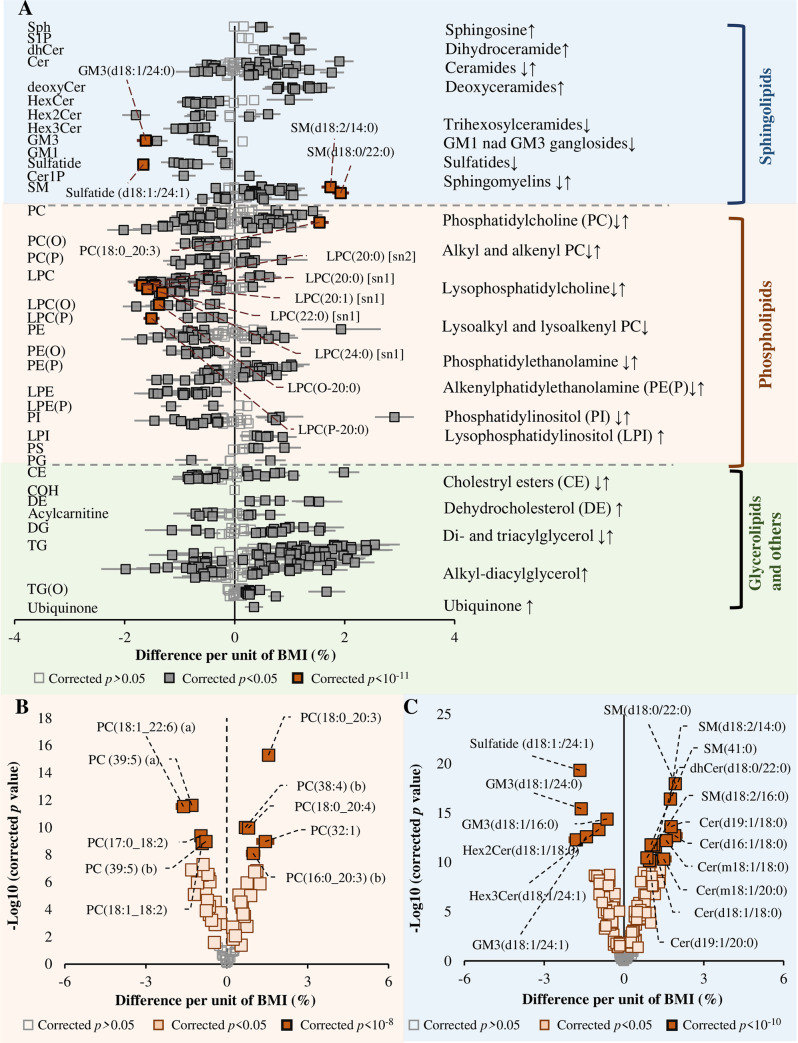
The association between BMI and the plasma lipidome. (A) Linear regression analysis between BMI and log-transformed lipid concentration was performed on 10,339 subjects adjusting for age, sex, total cholesterol, HDL-C, and triglycerides. Open squares show nonsignificant species, and grey and yellow closed squares show species with corrected *p* < 0.05 and species showing most significant associations (corrected *p* < 1 × 10^−11^), respectively. Error bars represent the 95% confidence interval. (B) The association of BMI with PC species and (C) the association of BMI with sphingolipid species. In panels B and C, the grey, pale tan, and dark tan squares indicate lipid species with corrected *p*-value greater than 0.05 or less than 0.05 and most significant, respectively. See S1 Data for the underlying data. BMI, body mass index; CE, cholesteryl ester; Cer, ceramide; Cer-1-P, ceramide-1-phosphate; COH, free cholesterol; DE, dehydrocholesterol; deoxyCer, deoxyceramide; DG, diacylglycerol; dhCer, dihydroceramide; GM1, G_M1_ ganglioside; GM3, G_M3_ ganglioside; HDL-C, high-density lipoprotein cholesterol; HexCer, monohexosylceramide; Hex2Cer, dihexosylceramide; Hex3Cer, trihexosylceramide; LPC, lysophosphatidylcholine; LPC(O), lysoalkylphosphatidylcholine; LPC(P), lysoalkenylphosphatidycholine; LPE, lysophosphatidylethanolamine; LPE(P), lysoalkenylphosphatidylethanolamine; LPI, lysophosphatidylinositol; PC, phosphatidylcholine; PC(O), alkylphosphatidylcholine; PC(P), alkenylphosphatidylcholine; PE, phosphatidylethanolamine; PE(O), alkylphosphatidylethanolamine; PE(P), alkenylphosphatidylethanolamine; PG, phosphatidylglycerol; PI, phosphatidylinositol; PS, phosphatidylserine; SM, sphingomyelin; sn, stereospecifically numbered; Sph, sphingosine; S-1-P, sphingosine-1-phosphate; TG, triacylglycerol; TG(O), alkyl-diacylglycerol.

Independent of age and sex, there were 577 lipid species associated with BMI (Fig 7A). In analyses adjusted for age, sex, total cholesterol, HDL-C, and triglycerides, 508 species were associated with BMI. Sphingolipids, including sphingosine, ceramide-1-phosphate, dihydroceramide, and deoxy-ceramide, were positively associated with BMI, whereas species of ganglioside, sulfatide, and mono-, di-, and trihexosylceramide were negatively associated (Fig 7A–7C). BMI was associated with most ceramide and sphingomyelin species, except those containing a relatively longer-chain fatty-acyl chain (Fig 7C). Most lysophospholipid species were negatively associated with BMI, as were ether-linked phospholipids (Fig 7A). Phosphatidylcholine species containing polyunsaturated omega-6 fatty acids such as 20:3, 20:4, or 22:4 were positively associated, whereas those containing the omega-6 fatty acid 18:2 and/or very long-chain omega-3 fatty acids were negatively associated (Fig 7B).

**Fig 8:**
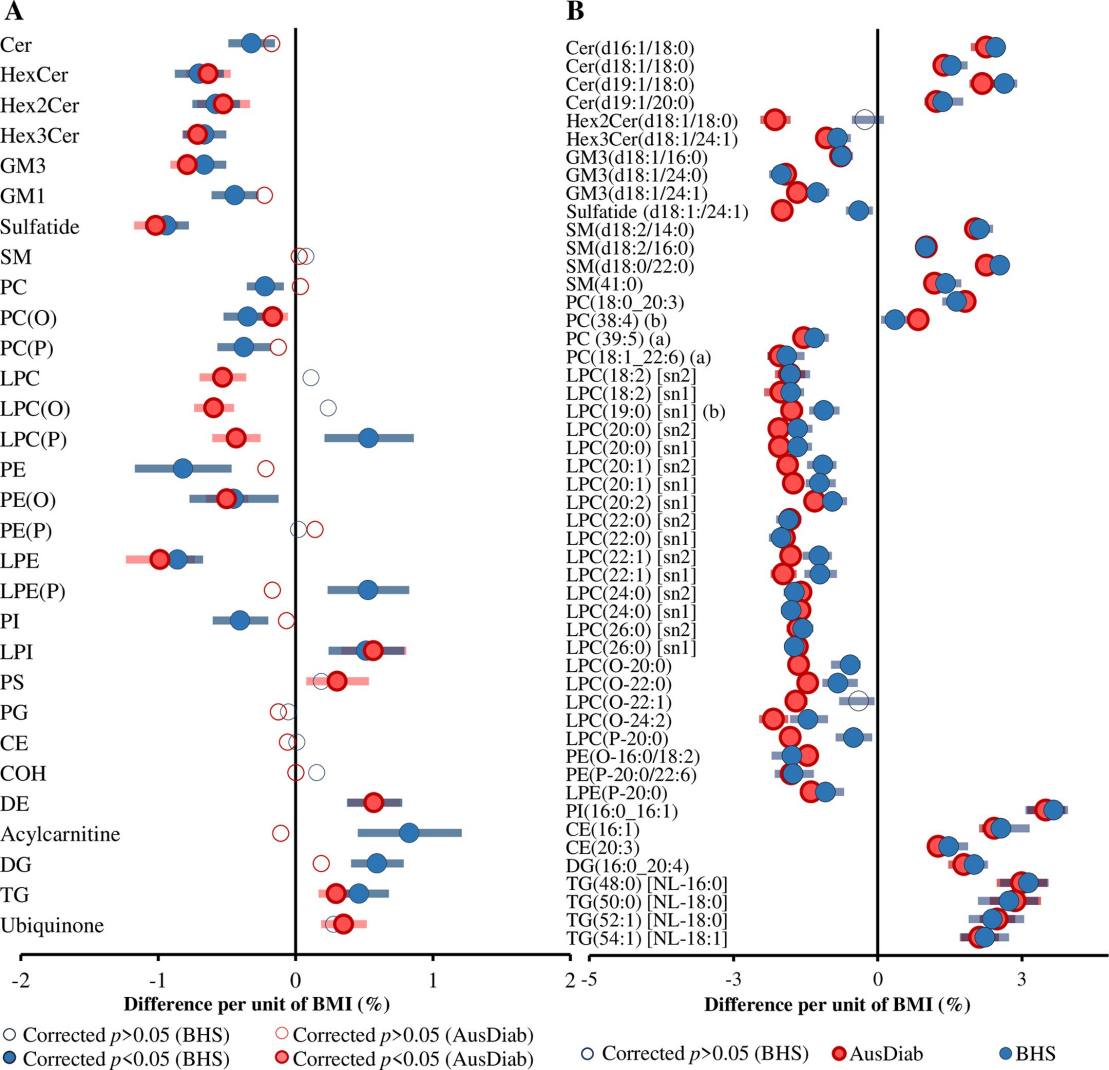
Validation of the association between BMI and plasma lipid classes/subclasses and species. (A) Linear regression analysis between BMI and log-transformed lipid class concentrations was performed on 10,339 AusDiab participants and 4,097 Busselton participants. (B) The same analyses were performed at the lipid species level, and the 50 most significant species in the AusDiab were selected based on lowest corrected *p*-values. The analyses were adjusted for age, sex, total cholesterol, HDL-C, and triglycerides. Pink and blue closed circles show lipid species/classes associated with BMI in AusDiab and Busselton cohort, respectively (*p* < 0.05). Open circles represent nonsignificant associations (corrected *p* > 0.05). Error bars represent the 95% confidence intervals. See S1 Data for the underlying data. AusDiab, Australian Diabetes, Obesity and Lifestyle Study; BMI, body mass index; CE, cholesteryl ester; Cer, ceramide; Cer-1-P, ceramide-1-phosphate; COH, free cholesterol; DE, dehydrocholesterol; deoxyCer, deoxyceramide; DG, diacylglycerol; dhCer, dihydroceramide; GM1, G_M1_ ganglioside; GM3, G_M3_ ganglioside; HDL-C, high-density lipoprotein cholesterol; HexCer, monohexosylceramide; Hex2Cer, dihexosylceramide; Hex3Cer, trihexosylceramide; LPC, lysophosphatidylcholine; LPC(O), lysoalkylphosphatidylcholine; LPC(P), lysoalkenylphosphatidycholine; LPE, lysophosphatidylethanolamine; LPE(P), lysoalkenylphosphatidylethanolamine; LPI, lysophosphatidylinositol; NL, neutral loss; PC, phosphatidylcholine; PC(O), alkylphosphatidylcholine; PC(P), alkenylphosphatidylcholine; PE, phosphatidylethanolamine; PE(O), alkylphosphatidylethanomine; PE(P), alkenylphosphatidylethanolamine; PG, phosphatidylglycerol; PI, phosphatidylinositol; PS, phosphatidylserine; SM, sphingomyelin; sn, stereospecifically numbered; TG, triacylglycerol.

Many lipid species and lipid subclasses/classes associated with BMI in the AusDiab cohort were replicated in the Busselton cohort. At the class/subclass level, 16 out of 30 were significantly associated with BMI in the AusDiab cohort, and of these, 11 were significant in the same direction, 4 were not significant, and 1 subclass, lysoalkenylphosphatidylcholine, showed an opposite association in the Busselton cohort (Fig 8A). In addition, a total of 337 out of 563 common lipid species were replicated in Busselton with the effect sizes in the same direction as in the AusDiab discovery cohort (S7 Table). Among the 50 species with the strongest association in the AusDiab cohort (*p* < 5.90 × 10^−11^), 48 were replicated in the Busselton cohort (Fig 8B). The correlation between regression coefficients of each lipid in the AusDiab and the Busselton cohort was strong (r^2^ = 0.750) (S10 Fig).

**Fig 9:**
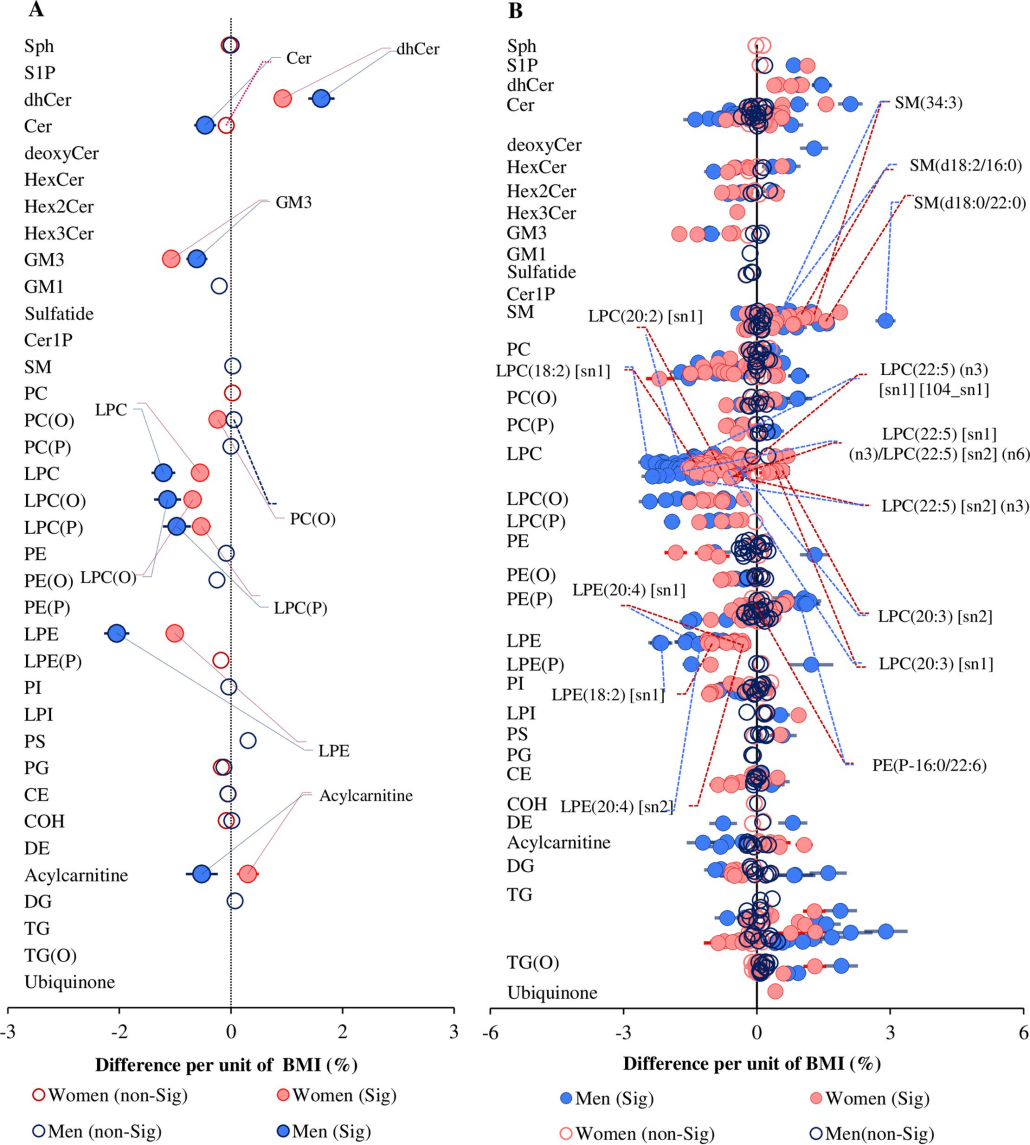
Sex-specific association between BMI and plasma lipid classes and species. (A) Linear regression analysis between BMI and log-transformed lipid class concentrations on 10,339 participants (men = 4,654 and women = 5,685) adjusting for age, sex, total cholesterol, HDL-C, and triglycerides and including a sex × BMI interaction term. (B) Linear regression analyses between BMI and log-transformed lipid species concentrations including sex interaction, adjusting for age, sex, total cholesterol, HDL-C, and triglycerides. Associations with lipid species that showed a significant sex interaction (corrected *p* < 0.05) were plotted separately for women (pink) and men (blue). Closed circles show significant associations (corrected *p* < 0.05). Open circles show corrected *p* > 0.05. Error bars represent 95% confidence intervals. See S1 Data for the underlying data. BMI, body mass index; CE, cholesteryl ester; Cer, ceramide; Cer-1-P, ceramide-1-phosphate; COH, free cholesterol; DE, dehydrocholesterol; deoxyCer, deoxyceramide; DG, diacylglycerol; dhCer, dihydroceramide; GM1, G_M1_ ganglioside; GM3, G_M3_ ganglioside; HDL-C, high-density lipoprotein cholesterol; HexCer, monohexosylceramide; Hex2Cer, dihexosylceramide; Hex3Cer, trihexosylceramide; LPC, lysophosphatidylcholine; LPC(O), lysoalkylphosphatidylcholine; LPC(P), lysoalkenylphosphatidylcholine; LPE, lysophosphatidylethanolamine; LPE(P), lysoalkenylphosphatidylethanolamine; LPI, lysophosphatidylinositol; PC, phosphatidylcholine; PC(O), alkylphosphatidylcholine; PC(P), alkenylphosphatidylcholine; PE, phosphatidylethanolamine; PE(O), alkylphosphatidylethanolamine; PE(P), alkenylphosphatidylethanolamine; PG, phosphatidylglycerol; PI, phosphatidylinositol; PS, phosphatidylserine; SM, sphingomyelin; sn, stereospecifically numbered; Sph, sphingosine; S-1-P, sphingosine-1-phosphate; TG, triacylglycerol; TG(O), alkyl-diacylglycerol.

We further tested whether the association between BMI and circulating lipid classes/subclasses and species was sex dependent. We fitted a linear model including an interaction term for sex, adjusted for age, sex, total cholesterol, HDL-C, and triglycerides. At the class/subclass level, the associations of BMI with ceramide, dihydroceramide, G_M3_ ganglioside, and several phospholipid classes/subclasses were significantly different between men and women (interaction *p* < 0.05). Acylcarnitines were positively associated in women but negatively associated in men (Fig 9A). Analyses based on individual lipid species resulted in a total of 323 lipid species showing a significant sex interaction (corrected interaction *p*-value < 0.05) (Fig 9B). The association of phospholipids in general and lysophospholipids in particular with BMI was typically different between men and women.

### Associations with waist circumference (WC) and waist/hip ratio (WHR)

BMI, WC, and WHR were correlated with each other (S11 Fig). We observed a similar association of lipids with BMI, WC, and WHR (S12 Fig). Of the 706 lipid species, some 357 lipid species were associated with all 3 measures (BMI, WC, and WHR). In addition, there were many other species associated with BMI and WC or BMI and WHR or WC and WHR. A summary of this overlapped association is outlined in S13 Fig. There were differences in the strength of some associations—triacylglycerol species tended to be more strongly associated with WC or WHR compared with BMI. Deoxyceramide and lysoalkylphosphatidylcholine species showed a stronger association with WHR than with BMI. There were 40, 30, and 9 lipid species associated with only BMI, WC, or WHR, respectively. The list of these lipids can be found in S13 Fig.

### Association of smoking with the plasma lipidome

In this study, smokers (*n* = 1,623) compared with nonsmokers (*n* = 5,632) had higher levels of saturated and monounsaturated fatty-acid–containing lipid species and lower levels of polyunsaturated fatty acid phospholipids and ether-linked lipid classes and subclasses, including alkylphosphatidylethanolamine and alkyl-diacylglycerol (S14 Fig).

### Association of risk factors with the fatty acid composition of the lipidome

Our current method provides full characterisation of the individual fatty-acyl chain composition for the majority of lipid species. This can be informative, particularly for lipid species containing only a single fatty acid (e.g., lysophosphatidylcholine) where the association may be more readily interpreted. To investigate this, we summed the sn1 and sn2 isomers for each lysophosphatidylcholine species and performed a correlation analysis. Using compositional data (lipids expressed as the percentage of their class total), we performed regression analyses with age, sex, and BMI. The lysophospholipid species (representing fatty acids) showed a strong positive correlation between odd-chain fatty acids and, to a lesser extent, between even-chain fatty acids (S15 Fig). Linear regression analyses, adjusted for sex, BMI, total cholesterol, HDL-C, and triglycerides showed that age was positively associated with long-chain to very long-chain fatty acids and fatty acids in the omega-3 pathway. Medium-chain and odd-chain fatty acids, including 14:0 and 15:0 and the omega-6 pathway, showed strong inverse association with age (S16A Fig). Regression analyses with sex adjusted for age, BMI, total cholesterol, HDL-C, and triglycerides showed most polyunsaturated fatty acids to be higher in men, whereas saturated and some monounsaturated fatty acids, including the C16:1 species, were lower in men (S16B Fig). Regression analyses with BMI, adjusted for age, sex, total cholesterol, HDL-C, and triglycerides, showed negative association between most fatty acids and BMI. However, a few fatty acids such as myristic acid (14:0) and palmitoleic acid (16:1) were positively associated (S16C Fig).

### The use of lipid ratios to define metabolic pathways

To gain further insight into the relationship of lipid metabolic pathways with age, sex, and BMI, we performed an unbiased association analyses of with all lipid ratios adjusted for covariates (278,631 lipid ratios). We utilised the *p*-gain value (lowest *p*-value of the lipid species used in the ratio divided by the *p*-value of the lipid ratio) to identify lipid ratios that were providing new information and considered the *p*-gain values significant if they exceeded 10 × the number of ratios tested (2.79 × 10^6^) as described by Petersen and colleagues [28]. There were 38,519 lipid ratios with a significant *p*-gain value for BMI (S8 Table). When these were sorted (based on *p*-gain values), we observed the top-ranked lipid ratios contained lipid species that were not clearly related in metabolic pathways [e.g., SM(34:3)/PC(17:0_18:2), *p*-gain 1.50 × 10^258^; SM(d18:2/14:0)/LPC(26:0)[sn1], *p*-gain 1.69 × 10^248^] and were likely the result of internal normalisation of technical variance in the data set. However, some lipid ratios did appear to relate through metabolic pathways [e.g., SM(d18:2/16:0)/SM(d18:1/16:0), *p*-gain 2.85 × 10^224^; PE(18:0_22:6)/PE(16:0_22:6), *p*-gain 6.82 × 10^213^] (S8 Table). In each case, we noted a high correlation between the lipid species within the ratios. We subsequently filtered the lipid ratios to those with a correlation coefficient >0.7 between the lipid species within the ratio and then again ranked by *p*-gain. This provided a shorter list of 560 lipid ratios in which the lipid species within each ratio were more closely related.

**Fig 10:**
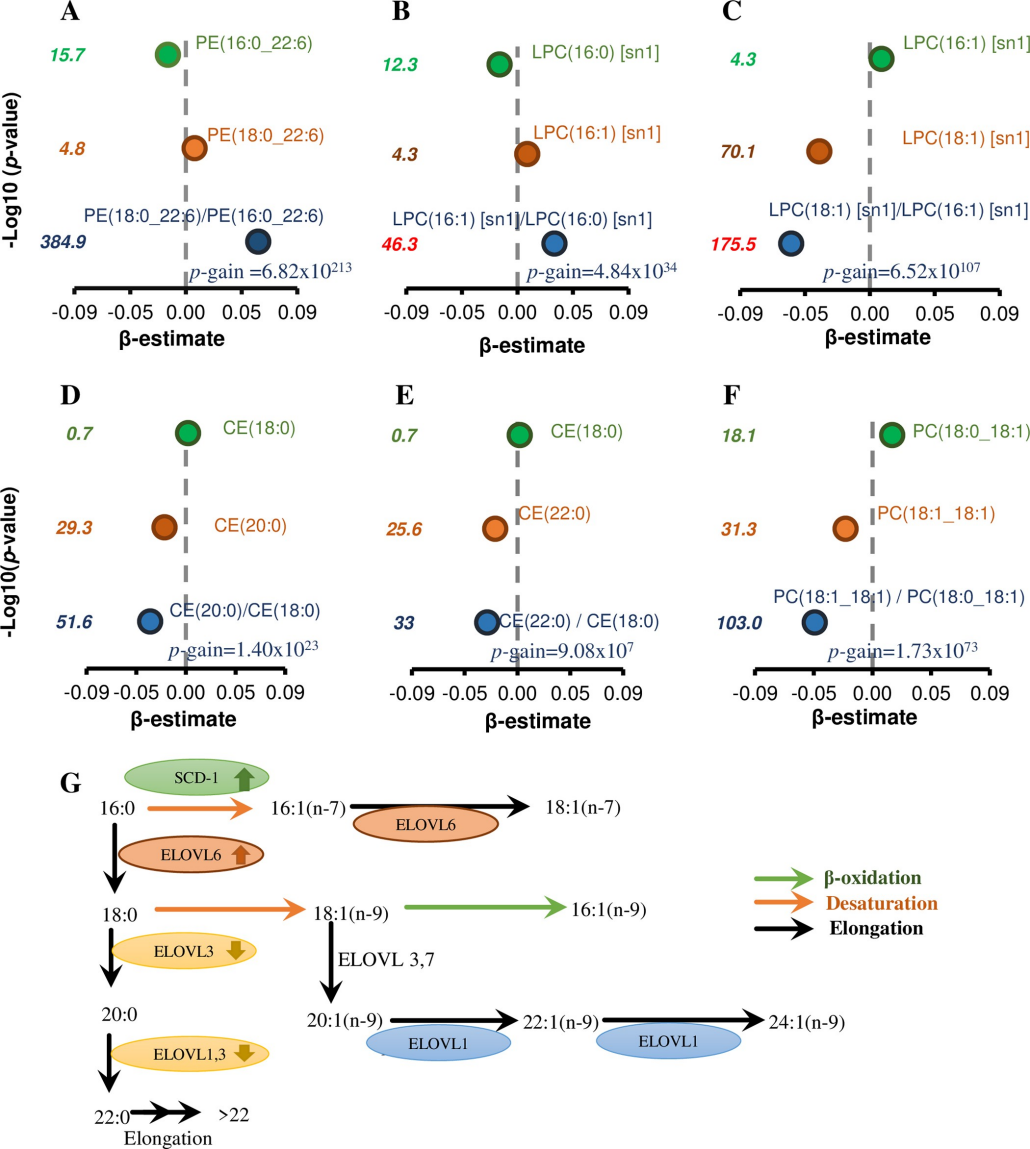
Plasma fatty acid ratios and enzyme pathways associated with BMI. A linear regression adjusted for age, sex, total cholesterol, HDL-C, and triglycerides was performed between individual lipid species or lipid ratios and BMI (panels A–F). The strength of associations with ratios relative to individual lipid species is indicated by *p*-gain values. (G) The de novo monounsaturated fatty acid pathway in mammals. The proposed increase or decrease in enzyme activities with increasing BMI is shown by arrows. BMI, body mass index; CE, cholesteryl ester; ELOVL, elongation of very long chain fatty acids protein; HDL-C, high-density lipoprotein cholesterol; LPC, lysophosphatidylcholine; PC, phosphatidylcholine; PE, phosphatidylethanolamine; SCD-1, stearoyl CoA desaturase 1; sn, stereospecifically numbered

Among the top-ranked ratios, we observed lipid pairs differing by specific fatty acids (e.g., 16:0/18:0; 16:0/16:1; 16:1/18:1) showing strong *p*-gain values in their association with BMI. We mapped some of these ratios to known metabolic pathways. We searched the data set for additional ratios in which the lipid species differed in a single feature (e.g., 16:0 to 18:0 or d18:1 to d18:2) and used this to identify supporting ratios for each of our biologically relevant top-ranked lipid ratios. The ratio of PE(18:0_22:6)/PE(16:0_22:6) (Fig 10A) was positively associated with BMI, as were 4 additional lipid ratios differing only in the 16:0 to 18:0 fatty acid (S9 Table). However, one ratio PI(18:0_20:4)/PI(16:0_20:4) was negatively associated with BMI, with a *p*-gain value of 5.87 × 10^11^ (S9 Table). Lipid ratios defined by a 16:1/16:0 fatty acid difference were also positively associated with BMI (Fig 10B), whereas the ratios defined by 18:1/16:1 (Fig 10C), 18:1/18:0 (Fig 10D), 20:0/18:0 (Fig 10E), and 22:0/18:0 (Fig 10F) were negatively associated with BMI. The mapping of the monounsaturated fatty acid pathway is shown in Fig 10G. Some of the same fatty acid pathways were also associated with age (S9 Table, S10 Table) and sex (S9 Table, S11 Table).

We also observed ratios of dihydroceramide/ceramide species (S17A Fig and S17B Fig), hexosylceramide/ceramide species (S17C Fig), and ceramide/sphingomyelin species (S17D Fig) associated with BMI that could be mapped to the sphingolipid biosynthetic pathway (S17 Fig, S12 Table). Ratios of sphingolipid species differing in their sphingoid base; e.g., d18:2 and d18:1-containing species, Hex2Cer(d18:2/16:0)/Hex2Cer(d18:1/16:0) (S17E Fig) and SM(d18:2/16:0)/SM(d18:1/16:0) (S17F Fig), were strongly associated with BMI and map to the fatty acid desaturase 3 (FADS3) (S17H Fig). The ratios of d18:2/d18:1 were also strongly associated with age (S13 Table) and women (S18A Fig). Similarly, ratios of alkenylphosphatidylethanolamine/alkylphosphatidylethanolamine species (e.g., PE(P-16:0/22:6)/PE(O-16:0/22:6)) were also strongly associated with BMI (S19 Fig, S14 Table), age, and sex (S14 Table) and signify the specific activity of plasmanylethanolamine Δ1-desaturase enzyme.

We have provided rationale and examples for our approach to mining the lipidomics data and provide full summary results of all lipid ratios with significant *p*-gain values for age, sex, and BMI as a reference tool and resource. We have also provided the correlation structures between lipid species in the entire cohort (S2 Fig, S1 Table), for men (S3 Fig, S2 Table), and for women (S4 Fig, S3 Table), as well as the differences in correlation coefficients (men relative to women) (S5 Fig, S4 Table).

## Discussion

This study is the first of its kind, to our knowledge, to examine over 700 circulating molecular lipid species in a large population-based cohort using a targeted lipidomics approach [16]. We demonstrated complex associations of the plasma lipidome with age, sex, and BMI, as well as with WC and WHR and smoking, and have mined these data to identify lipid metabolic pathways. We have further shown that the association between the plasma lipidome and age or BMI were sex- dependent. These findings provide the basis for understanding the dysregulation of lipid metabolism in aging and obesity and how this is influenced by sex. Many of the results were replicated in the BHS cohort, an independent cohort composed of participants with a comparable age range and ethnicity to the discovery cohort.

### The plasma lipidome differs between men and women

Sphingolipids are structurally diverse lipids characterised by a sphingoid backbone, the most abundant base in humans being the 18-carbon sphingosine (d18:1) accounting for 57%, followed by sphingadienine (d18:2, 21%) [29]. Sphingolipids containing the d18:2 sphingoid base showed strong associations with sex (higher levels in women). The dienic base contains an extra double bond at *cis*-14 [30] in addition to the 4E double bond, giving rise to d18:2 (4E,14Z). The enzyme responsible for the incorporation of this extra double bond was recently reported as FADS3 [29]. They reported a higher level of d18:2 sphingomyelin species (30%) in women relative to men and a corresponding higher level of FADS3 activity [29]. We performed regression analysis of lipid ratios with sex. By filtering the ratios based on the correlation between the lipid species and sorting based on the *p*-gain, we identified biologically relevant lipid ratios that captured the same FADS3 signal (S18B Fig). The association of sex with SM(d18:2/24:0) and SM(d18:1/24:0) had *p*-values of 4.26 × 10^−7^ and 2.28 × 10^−49^, respectively, whereas the ratio of SM(d18:2/24:0)/SM(d18:1/24:0) had a *p*-value of 1.26 × 10^−139^; *p*-gain = 2.80 × 10^91^, clearly reflecting the differential activity of the FADS3 enzyme in men and women.

Atypical sphingolipids that arise from noncanonical sphingolipid pathways, such as the 1-deoxyceramide species, were higher in men than in women. Deoxyceramide is synthesised from the condensation of palmitoyl-CoA with alanine by the action of serine palmitoyltransferase (SPT), leading to the formation of atypical ceramides lacking a hydroxyl group at the C1 position (53). Phosphorylation of the C1 hydroxyl group is requisite for catabolism of sphingolipids. Thus, it is not clear how such metabolites are catabolised, leading to the speculation they may accumulate throughout life. It is, unclear at this point whether difference in substrates (alanine/serine) levels as previously reported [31] or a difference in rate of synthesis and/or turnover is responsible for the observed variation in the levels of deoxyceramide species between men and women. In disease conditions, deoxyceramide species have emerged as biomarkers of metabolic syndrome and T2D [32, 33]. Gender- and age-related differences (also observed in this study) have been reported in the risk, pathophysiology, and complications of T2D [22, 34]. Our findings highlight the possibility that dysregulation in atypical sphingolipid metabolism with age and sex may contribute to cardiometabolic risk.

Lysophosphatidylcholine and ether-linked phospholipids were significantly elevated in the plasma of men compared with women, suggesting there is a sexual dimorphism in phospholipid metabolism, which could be partly due to differences in the activity of phospholipases and/or complex hormonal, dietary, and lifestyle factors. Two phospholipid-metabolising enzymes, lecithin cholesterol acyltransferase (LCAT) [35] and lipoprotein-associated phospholipase A2 (PLA2) [36], have been reported to differ between men and women and likely contribute to these lipidomic associations. Indeed, increased activity and/or mass of lipoprotein-associated PLA2 is an independent risk factor for stroke and cardiovascular disease [37, 38].

### The plasma lipidome is associated with age

The positive association of acylcarnitine species with age, independent of sex, BMI, and clinical lipids is a key finding of our study. Huynh and colleagues have reported a similar finding [16]. The elevated circulating acylcarnitine species may reflect impaired mitochondrial β-oxidation [39], which has previously been associated with age [40, 41]. In a mice model, Bodil and colleagues have reported an increase in plasma acylcarnitine levels caused by inhibition of mitochondrial fatty acid oxidation [42]. In clinical studies, elevated plasma acylcarnitine levels have been shown to be associated with age-related cardiometabolic conditions such as the risk of T2D and CVD, independently of age [43–46]. Notwithstanding these observations, other explanations are possible, and further studies are required to validate our findings.

A striking finding was the negative association between age and most ether lipids, including alkylphosphatidylcholine, alkenylphosphatidylcholine, alkylphosphatidylethanolamine, and ether-linked triacylglycerol species. Reduced levels of ether lipids, particularly plasmalogens, have been associated with oxidative stress and age-related metabolic disorders [47, 48]. Lipidomic studies have reported that plasmalogens are inversely associated with obesity, T2D, CVD, and Alzheimer’s disease [14, 49, 50]. Chaleckis and colleagues demonstrated that the levels of antioxidant compounds were reduced in elderly compared with young adults [51], and so higher levels of oxidative stress may contribute to oxidation and turnover of plasmalogens or species containing polyunsaturated fatty-acyl groups, which are both susceptible to oxidation. We also observed multiple species of ether-phospholipids (alkylphosphatidylcholine and alkylphosphatidylethanolamine) and ether glycerolipids (alkyl-diacylglycerol) that did not contain polyunsaturated acyl chains negatively associated with age. This suggests an alternative hypothesis: down-regulation of the de novo synthesis resulting from peroxisomal dysfunction with aging. This concept has been proposed previously [52, 53] and is based primarily on the associations between peroxisomal dysfunction as measured by decreased plasmalogens and other peroxisomal-derived lipids and age-related diseases such as diabetes, CVD, and Alzheimer’s disease [14, 54, 55].

Interestingly, we observed that some ether-phospholipids containing n3 fatty acyls, including docosahexaenoic acid (DHA) and EPA, were positively associated with age, possibly as a result of higher dietary intake in the older population as has been reported [56]. However, whilst omega-3 supplementation can increase these species of ether lipids, it does not increase the total ether lipid content of plasma and so is unlikely to attenuate the peroxisomal driven defects associated with aging [57]. Such peroxisomal defects are also supported by the strong positive association of age with saturated and monounsaturated very long-chain fatty acids, including 22:1, 24:0, and 26:0, which are metabolised via β-oxidation exclusively in peroxisomes. In line with our findings, elevated 24:0 fatty acid levels with age have also been documented [58]. The effect of diet on omega-3 fatty acid levels was further supported by the fatty-acyl side-chain–specific analyses, in which fatty acids of the n3 pathway, including 20:5 and 22:6 (DHA) as well as the 22:5(n3), were positively associated with age, whereas the n-6 fatty acid species 20:3, 22:4, and 22:5(n6) showed a negative association with age. Elongation of very long chain fatty acids protein (*ELOVL2*) and *ELOVL5* genes play a key role in the biosynthesis of very long-chain polyunsaturated fatty acids. However, the recent report of a methylation-induced decrease in the expression of *ELOVL2* with age [59], combined with the opposing associations of the n3 and n6 fatty acids, would suggest the n3 increases observed here were not due to the up-regulation of the biosynthetic pathway, but rather a dietary effect.

### The association between age and the plasma lipidome is sex-dependent

In sex-stratified analysis, we observed a clear difference in lipidomic profiles across age groups in men and women. These results suggest that there could be a differential impact of aging on lipid metabolism in men compared to women; understanding these complex interactions will help identify sex-specific lipidomic biomarkers for more personalised interventions. In women, the age fingerprint was marked by a shift starting at about 48 years of age (coinciding with menopause) as opposed to an earlier shift (at about 38 years) in men.

Profiles of lysophosphatidylcholine, lysoalkylphosphatidylcholine, lysoalkenylphosphatidylcholine, alkylphosphatidylcholine, alkylphosphatidylcholine, alkylphosphatidylethanolamine, and alkenylphosphatidylethanolamine with age were particularly different between men and women. These lipids showed a stronger negative association with age in men relative to women, suggesting that age has a differential effect on lyso- and ether-phospholipid metabolism based on sex. As discussed earlier, hydrolysis of phosphatidylcholine by the enzymatic action of PLA2 and LCAT results in higher levels of lysophosphatidylcholine in men. However, it is not clear whether there is a greater decrease in PLA2 and LCAT activity or increased clearance of lysophosphatidylcholine with age in men compared to women.

Lysophospholipids containing omega-3 fatty acids such as DPA (22:5n-3) showed the strongest age–sex interaction (being negatively associated with age in men and positively associated in women). This may reflect differences in the omega-3 synthetic pathway to produce 22:5n-3 [60], in diet, or in both.

Our findings also suggest that aging has a differential effect on peroxisomal ether-phospholipid biosynthesis in men and women. Ether lipids including the alkyl-ether and plasmalogens (alkenyl-ethers) are subclasses of glycerophospholipids that have the same initial step in their biosynthesis [47]. Here, we demonstrated age to be associated with ether-phospholipids, particularly plasmalogens such as alkenylphosphatidylethanolamine and alkenylphosphatidylcholine, differently in men and women. This strong age and sex effect on the circulating plasmalogens suggests that specific mechanisms exist that regulate ether-phospholipid biosynthetic pathway differently between men and women based on their age. Whilst aging has been shown to be associated with a substantial reduction in plasmalogens [61], this has not been shown to be sex-specific. Whether age- and sex-related differences in the action and/or expression of enzymes involved in ether-phospholipid biosynthesis exist or whether sex hormones play a role in driving these differences needs to be further investigated.

### Menopausal status is associated with the lipidomic profile

We observed a significant difference in the lipidomic profile between post- and premenopausal women. Menopause is accompanied by hormonal changes, which in turn are associated with increased abdominal fat and risk of cardiometabolic diseases [62, 63]. Menopause status has been reported to contribute to the rise in risk of CVD, mainly due to changes in atherogenic lipids [64], oxidative stress [65], or endothelial dysfunction in women [66]. Elevated levels of traditional plasma lipid measures including total cholesterol, LDL-C, and triglycerides have been reported in association with menopause [67, 68]. The higher levels of phosphatidylcholine, phosphatidylinositol, sphingomyelin, phosphatidylethanolamine, ceramide, and di- and triacylglycerol species observed in postmenopausal women in the present study may reflect the effect of hormonal changes during menopause on liver lipoprotein metabolism [69]. Previous studies have shown that menopause and abdominal obesity are major determinants of hepatic lipid metabolism [70], and thus, it is not surprising to observe plasma lipidomic changes in relation to menopause. In agreement with our findings, a menopause-associated increase in atherogenic lipoprotein profile, including plasma total cholesterol, LDL-C, triglycerides, very low-density lipoprotein cholesterol (VLDL-C), and apolipoprotein B (ApoB) [27, 67, 68] as well as phosphatidylcholine [27], has been reported. We have also observed an inverse association of alkyl chain containing ether-phospholipids, particularly alkylysophosphatidylcholine, alkylphosphatidylcholine, and alkylphosphatidylethanolamine species. Decreased levels of ether-phospholipids in conditions of oxidative stress, including Alzheimer’s disease [49], T2D [14], and CVD [71] have been documented. Menopause may contribute to neurodegenerative and cardiometabolic risk by modulating the metabolism of ether-phospholipids via either impaired liver lipid metabolism or peroxisomal dysfunction.

### The association of BMI and smoking with the plasma lipidome

BMI is the most widely used measure to define obesity and is a risk factor for multiple cardiometabolic diseases, including fatty liver disease and T2D. Here, we report BMI associated with 577 lipid species in age- and sex-adjusted models and 508 lipid species in age-, sex-, and clinical lipids (total cholesterol, HDL-C, and triglyceride)-adjusted models. Adjustment for clinical lipids serves to remove the associations driven by the changes in circulating lipoprotein levels (increased VLDL and LDL and decreased HDL) and so reveals the underlying associations with lipid metabolism. Smoking is a potential confounder of the association of BMI with lipid species [72]. However, adjustment for smoking had little effect on these associations.

In the current study, complex glycosphingolipids, including mono-, di-, and trihexosylceramide as well as G_M3_ ganglioside and sulfatide species, showed negative associations, whereas sphingomyelin, dihydroceramide, and deoxyceramide showed positive associations with BMI. These associations confirm and expand on our and others earlier reports of lipid species associated with BMI [16, 19, 73]. Examination of the associations of BMI with lipid ratios revealed highly significant *p*-gain values for many lipid ratios. Of note, the *p*-gain value for the lipid ratio SM(d18:2/16:0)/SM(d18:1/16:0) was 2.85 × 10^224^, suggesting an up-regulation of FADS3 activity with increasing BMI. This highlights the interaction of sex with lipid metabolism because FADS3, which converts d18:1 sphingolipids to d18:2, is expressed in higher levels in women (defined by the lipid ratio SM(d18:2/24:0)/SM(d18:1/24:0)) and as reported previously [29].

LPC species were negatively associated with BMI, with the exception of 14:0 or 16:1 fatty-acid–containing species, which showed positive associations. The negative association of LPC with BMI could be due to increased PLA2 activity. A study examining over 1,000 metabolites by Cirulli and colleagues showed a strong metabolome perturbation was associated with BMI and metabolic risk and identified several lysophosphatidylcholine species showing the same direction of association as observed in this study [74]. The positive associations observed for LPC(14:0) and LPC(16:1) likely reflect the increased de novo fatty acid synthesis associated with BMI that produces 14:0 and 16:1 fatty acids. This was highlighted in analyses with the fatty acid composition of the lysophosphatidylcholine species, in which these species showed the strongest positive association with BMI. Lipogenesis and the subsequent metabolism of fatty acids was also captured in the lipid ratio analyses. The strength of the association of BMI with (16:1/16:0) compared with either of the 2 fatty acids, e.g., signifies a biological role of the stearoyl CoA desaturase 1 (*SCD-1*) enzyme that converts 16:0 fatty acid to 16:1 fatty acid [75]. Increased activity of *SCD-1* has been associated with obesity and insulin resistance [76]. The ratio of 16:1/16:0 has also been associated with waist gain and long-term risk of metabolic syndrome [77]. We also observed certain saturated fatty acid ratios strongly associated with BMI. The conversion of the fatty acid 16:0 to 18:0, captured in the lipid ratio PE(18:0_22:6)/PE(16:0_22:6) (*p*-gain 6.82 × 10^213^) was strongly associated with BMI and likely represents an up-regulation of *ELOVL6* activity. Analyses of related lipid ratios further suggests a down-regulation of *ELOVL1* and/or *ELOVL3* as depicted in Fig 10. However, not all lipid ratios provided clear signals of enzyme activities; the ratio PC(18:0_18:1)/PC(18:1_18:1), which might also be thought to capture the SCD-1 activity, indicated a down-regulation of the conversion of 18:0 to 18:1, in contradiction to the 16:0 to 16:1 conversion. As indicated in Fig 10, other sources of 18:1, including diet, and turnover of 18:1 may also impact these ratios and confound biological interpretation. Thus, caution must be exercised in such interpretations.

One clear lipid ratio signal associated with BMI was the ratio between alkenylphosphatidylethanolamine/alkylphosphatidylethanolamine species; e.g., the PE(P-16:0/22:6)/PE(O-16:0/22:6) ratio displayed a *p*-gain of 2.57 × 10^33^, which suggests the up-regulation of the plasmanylethanolamine delta 1-desaturase enzyme, responsible for the introduction of a characteristic vinyl ether double bond into plasmalogens, with increasing BMI [78]. Notwithstanding the limitations described above, our findings highlight the potential of lipid ratios to identify enzymatic pathways being altered in response to changes in adiposity and other metabolic traits. Our results agree with the findings of previous studies reporting that the strength of associations with metabolic phenotypes increases when using metabolite ratios rather than individual metabolites [79].

In the present study, we found a negative association between BMI and lysophosphatidylcholine, alkylphosphatidylcholine, alkenylphosphatidylcholine, and lysophosphatidylethanolamine; these associations were stronger in men than in women. A study by Gerl and colleagues revealed similar findings [80]; in particular, the stronger negative association of lysophospholipids with BMI in men compared with women was consistent with our findings. Acylcarnitine species showed contrasting associations with BMI based on sex (i.e., negative association in men and positive association in women), suggesting a sex-specific regulation of acylcarnitine. Men and women have been shown to differ in many metabolic aspects; body fat distribution and muscle mass represent well-known contributors to sex differences [81, 82], and these may drive the differential regulation of acylcarnitine and energy metabolism.

Finally, we show that smoking had a strong effect on the plasma lipidome. Our observations of negative associations with lipids containing polyunsaturated fatty acids, particularly omega-3 species such as DHA (C22:6), and positive association with saturated or monounsaturated species agree with previous reports [83, 84]. Smoking modulates essential fatty acid metabolism and results in a reduction of polyunsaturated fatty acid levels through depletion of antioxidants and subsequent increase in lipid peroxidation [85]. These changes in fatty acid metabolism may in turn relate to the significant reduction in HDL-C [86] and the shift towards an atherogenic lipid profile [83, 87].

### Comparison with commercial lipidomics solutions

The targeted lipid panel reported in this study constitutes 706 distinct lipid species that span 36 lipid classes/subclasses. For 506 of these, we provide alkyl, alkenyl, and acyl-chain resolution (e.g., PE(P-18:0/20:4)), whereas only 83 species are reported as the sum totals (e.g., PC(38:2)), with the remainder showing some level of fatty acid definition (e.g., TG(48:2) [neutral loss (NL)-14:0]). Comparable platforms that are able to perform larger cohort studies include the Biocrates AbsoluteIDQ p180 kit, which has been used extensively to provide data on multiple large cohort studies [88, 89] but covers a smaller number of lipid classes, with limited structural resolution owing to technical limitations [90, 91]. However, we do observe general alignment with our findings; Trabado and colleagues measured 185 plasma metabolites in 924 healthy individuals using the AbsoluteIDQ p180 kit and showed higher lysophosphatidylcholine and lower sphingomyelin and phosphatidylcholine species in men relative to women [91]. More recent expanded platforms such as the Biocrates MxP Quant 500 platform and the SCIEX Lipidyzer platform have provided expanded coverage of the lipidome. Although structural resolution is still limited on the MxP Quant 500 platform, greater structural resolution of lipid species is available from the SCIEX Lipidyzer platform. Larger cohorts using these more recent platforms have not yet been reported.

Here, we provide machine-readable lipid identifiers for 91.3% of the lipid species from the SwissLipids database to facilitate comparison of our results with other studies (S15 Table).

### Limitations and strengths of the study

The major strengths of this study are 1) the large population-based sample size of the cohort, comprising over 10,000 participants in the discovery cohort and over 4,000 in the replication set and 2) improved lipidome coverage (over 700 species across 36 classes/subclasses). This study now provides a powerful resource for further lipidomic studies. The major limitation is, however, the cross-sectional nature of the present study, which did not allow us to determine causality of the relationships. In addition, the response rate of the AusDiab cohort was relatively low; hence further studies are needed to prove the generalisability our findings to other population groups and ethnicities. Despite the limitations, our study has uncovered novel, to our knowledge, associations of lipidomic biomarkers with common cardiometabolic risk factors, thus improving our current understanding of lipid biology. The sex-specific nature of the association of the lipidome with age and BMI may underpin the sex differences in the pathogenesis of age-related cardiometabolic diseases.

Our findings pave the way for further evaluation of the associations of plasma lipidomic profiles with disease risk factors in a sex-specific manner. We suggest that sex plays an important role in lipid metabolism associated with cardiometabolic risk, and understanding this will be essential for sex-specific biomarker discovery and precision medicine. Indeed, it is important to consider age- and sex-specific stratification during the design and analysis of cohort studies involving lipidomics.

## Materials and methods

### Study cohorts

#### AusDiab

We utilised all baseline fasting plasma samples from the AusDiab cohort (*n* = 10,339). The AusDiab cohort is the largest population-based prospective population study that was established to study the prevalence and risk factors of diabetes and CVD in the Australian adult population. The baseline survey was conducted in 1999–2000, with 11,247 participants aged ≥25 years from randomly selected areas from the 6 states and the Northern Territory, comprising 42 urban and rural areas of Australia, using a stratified cluster sampling method. The detailed description of study population, methods, and response rates of the AusDiab cohort is found elsewhere [92]. Measurement techniques for clinical lipids, including fasting serum total cholesterol, HDL-C, and triglycerides, as well as for height, weight, BMI, and other behavioural risk factors, have been described previously [93]. Here, we performed a comprehensive plasma lipidomic analysis on a total of 10,358 baseline fasting plasma samples after excluding samples from pregnant women (*n* = 19), those with missing data (*n* = 279), or those whose fasting plasma samples were unavailable or of inadequate amount for lipid extraction (*n* = 591). Following lipidomic analysis, additional samples (*n* = 19) were excluded from the final analysis because of technical issues. Thus, 10,339 participants (5,229 [51%] women) were included in the present analysis. The mean (range) age was 49 (25–91) and 47 (25–95) for women and men, respectively. The baseline characteristics of participants can be found in supplementary file (S16 Table).

#### Busselton study cohort

A total of 4,492 participants in the 1994/95 survey of the ongoing epidemiological study (the BHS) were included. The BHS is a population-based study in the town of Busselton, Western Australia; the participants are predominantly of European origin. The brief description of study subjects is found elsewhere (S16 Table). The details of the study characteristics and measurements for HDL-C, LDL-C, triglycerides, total cholesterol, and BMI are described elsewhere [94].

#### Ethics statement

This study used data sets from the AusDiab biobank (project grant APP1101320) approved by the Alfred Human Research Ethics Committee, Melbourne, Australia (project approval number, 41/18) and the BHS cohort (informed consent obtained from all participants, and the study was approved by the University of Western Australia Human Research Ethics Committee [UWA HREC; approval number, 608/15]). Both studies were conducted in accordance with the ethical principles of the Declaration of Helsinki.

#### Lipid extraction

Lipid extraction and analysis was carried out in a total of 28 batches; each run batch comprised 384 patient samples, 26 technical quality control (TQC) samples, 21 plasma quality control (PQC) samples, and 8 National Institute of Standards and Technology (NIST) samples. For each batch, a robot-assisted lipid extraction was carried out using the butanol/methanol method; the details are as described previously [95]. Briefly, 10 μL of plasma was mixed with 100 μL of butanol/methanol (1:1) with 10 mM ammonium formate. A standard mix containing some 22 internal standards (Table 1) representing 36 lipid classes were also included in the extraction solvent. The list of all internal standards is found in supplementary file (S17 Table). Samples were vortexed thoroughly, followed by sonication for 60 min at room temperature. Each sample was then centrifuged (14,000 × *g*, 10 min, 20°C). After centrifugation is complete, the supernatant containing the crude lipid extract was collected and transferred into Teflon glass vials with glass inserts.

**Table 1:** LC-MS/MS instrument conditions for targeted lipidomic analysis.

Lipid Class/Subclass	Parent Ion	Fragmentation	No. of Lipid Species	ISTD	ISTD (pmol)/Sample	Collision Energy (V)
Sphingosine	[M + H]+	NL, 18.0 Da	4	Sph(d17:1)	20	8
Sphingosine-1-phosphate	[M + H]+	sphingoid-base specific	4	Sph(d17:1)	25	10
Dihydroceramide	[M + H]+	sphingoid-base specific	6	dhCer(d18:0/8:0)	50	31
Ceramide	[M + H]+	sphingoid-base specific	50	Cer(d18:1-d7/17:0)	50	25
Deoxyceramide	[M + H]+	sphingoid-base specific	11	Cer(d18:1/17:0)	50	25
Monohexosylceramide	[M + H]+	sphingoid-base specific	14	HexCer (d18:1/16:0) d3	50	33
Dihexosylceramide	[M + H]+	sphingoid-base specific	10	Hex2Cer(d18:1/16:0) d3	50	53
Trihexosylcermide	[M + H]+	PI, m/z 264.3	6	Hex3Cer(d18:1/17:0)	25	57
G_M3_ ganglioside	[M + H]+	sphingoid base	8	Hex3Cer(d18:1/17:0)	25	57
G_M1_ ganglioside	[M + 2H]2+	PI, m/z 366.2	1	Hex3Cer(d18:1/17:0)	25	9
Sulfatide	[M + H]+	PI, m/z 264.3	10	sulfatide(d18:1/12:0)	10	56
Ceramide-1-phosphate	[M + H]+	PI, m/z 264.3	1	Cer(d18:1/17:0)	50	29
Sphingomyelin	[M + H]+	PI, m/z 184.1	44	SM(d18:1/12:0)	100	25
Phosphatidylcholine	[M + H]+	PI, m/z 184.1	70	PC(13:0/13:0)	100	21
Alkylphosphatidylcholine	[M + H]+	PI, m/z 184.1	22	PC(13:0/13:0)	100	21
Alkenylphosphatidylcholine	[M + H]+	PI, m/z 184.1	26	PC(13:0/13:0)	100	21
Lysophosphatidylcholine	[M + H]+	PI, m/z 184.1 and m/z 104.1	61	LPC(13:0)	100	21
Lysoalkylphosphatidylcholine	[M + H]+	PI, m/z 104.1	10	LPC(13:0)	100	21
Lysoalkenylphosphatidylcholine	[M + H]+	PI, m/z 104.1	6	LPC(13:0)	100	21
Phosphatidylethanolamine	[M + H]+	NL, 141.0 Da	37	PE(17:0/17:0)	100	17
Alkylphosphatidylethanolamine	[M + H]+	NL, 141.0 Da	14	PE(17:0/17:0)	100	17
Alkenylphosphatidylethanolamine	[M + H]+	acyl-chain specific	54	PE(17:0/17:0)	100	17
Lysophosphatidylethanolamine	[M + H]+	NL, 141.0 Da	14	PE(17:0/17:0)	100	17
Lysoalkenylphosphatidylethanolamine	[M + H]+	NL, 171.9 Da	4	PE(17:0/17:0)	100	19
Phosphatidylinositol	[M + NH4]+	NL, 277.0 Da	27	PE(17:0/17:0)	100	17
Lysophosphatidylinositol	[M + NH4]+	NL, 277.0 Da	8	LPI(13:0)	20	17
Phosphatidylserine	[M + H]+	NL, 185.0 Da	7	PS(17:0/17:0)	50	25
Phosphatidylglycerol	[M + NH4]+	NL, 189.0 Da	4	PG(17:0/17:0)	50	21
Free cholesterol	[M + NH4]+	PI, m/z 369.3	1	COH d7	10,000	23
Dehydrocholesterol	[M + NH4]+	PI, m/z 369.4	4	CE(18:0) d6	1,000	23
Cholesteryl ester	[M + NH4]+	PI, m/z 369.3	28	CE(18:0) d6	1,000	10
Acylcarnitines	[M + H]+	PI, m/z 85.1	14	Acylcarnitine(16:0) d3	10	30
Diacylglycerol	[M + NH4]+	NL, fatty acid	25	DG(15:0/15:0)	200	21
Triacylglycerol	[M + NH4]+	NL, fatty acid	112	TG(17:0/17:0/17:0)	100	21
Alkyl-diacylglycerol	[M + NH4]+	NL, fatty acid	28	TG(17:0/17:0/17:0)	100	21
Ubiquinone	[M + NH4]+	PI, m/z 197.0	1	Hex3Cer(d18:1/17:0)	50	21

**Abbreviations:** CE, cholesteryl ester; Cer, ceramide; Cer-1-P, ceramide-1-phosphate; COH, free cholesterol; DE, dehydrocholesterol; deoxyCer, deoxyceramide; DG, diacylglycerol; dhCer, dihydroceramide; GM3/1, G_M3_/G_M1_ ganglioside; HexCer, monohexosylceramide; Hex2Cer, dihexosylceramide; Hex3Cer, trihexosylceramide; ISTD, internal standard; LC-M/MS, liquid chromatography tandem mass-spectrometry; LPC, lysophosphatidylcholine; LPC(O), lysoalkylphosphatidylcholine; LPC(P), lysoalkenylphosphatidylcholine; LPE, lysophosphatidylethanolamine; LPE(P), lysoalkenylphosphatidylethanolamine; LPI, lysophosphatidylinositol; NL, neutral loss; PC, phosphatidylcholine; PC(O), alkylphosphatidylcholine; PC(P), alkenylphosphatidylcholine; PE, phosphatidylethanolamine; PE(O), alkylphosphatidylethanolamine; PE(P), alkenylphosphatidylethanolamine; PG, phosphatidylglycerol; PI, phosphatidylinositol; PS, phosphatidylserine; SM, sphingomyelin; Sph, sphingosine; S-1-P, sphingosine-1-phosphate; TG, triacylglycerol; TG(O), alkyl-diacylglycerol.

### LC-MS analysis

Lipidomic analysis was performed using LC electrospray ionisation MS/MS (LC-ESI-MS/MS). An Agilent 6490 triple quadrupole (QQQ) mass spectrometer (Agilent 1290 series HPLC system and a ZORBAX eclipse plus C18 column [2.1 × 100 mm × 1.8 μm; Agilent, Santa Clara, CA, USA]) in positive ion mode was used (details of the method and chromatography gradient have been described previously [16]). The solvent system consisted of solvent A, 50% H_2_O/30% acetonitrile/20% isopropanol (v/v/v) containing 10 mM ammonium formate, and solvent B, 1% H_2_O/9% acetonitrile/90% isopropanol (v/v/v) containing 10 mM ammonium formate. We used a linear gradient with a 14-minute cycle time and a 1-μL sample injection per sample. The following mass spectrometer conditions were used: gas temperature, 150°C; gas flow rate, 17 L/min; nebuliser, 20 psi; sheath gas temperature, 200°C; capillary voltage, 3,500 V; and sheath gas flow, 10 L/min. Given the large sample size, samples were run across several batches, as described above.

### Quality control

As part of monitoring the quality of sample extraction and LC-MS/MS analysis, PQC samples consisting of a pooled set of 10 healthy individuals were incorporated into the analysis at a rate of 1 PQC per 20 plasma samples. Additional, quality control samples utilising the NIST 1950 reference plasma sample (obtained from NIST, Gaithersburg, MD, USA) were inserted every 40 patient samples to facilitate future alignment with other studies. TQC samples consisted of PQC sample extracts that were pooled and split into individual vials to provide a measure of technical variation from the mass spectrometer only. These were included at a ratio of 1 TQC/20 plasma samples. TQCs were used to monitor for changes in peak area, width, and retention time to determine the performance of the LC-MS/MS analysis.

### Lipid quantitation and statistical analysis

Chromatographic peaks for each lipid were integrated using the Mass Hunter (B.07.00, Agilent Technologies) software. Relative quantification of lipid species was determined by comparing the peak areas of each lipid in each patient sample with the relevant internal standard (Table 1). Whilst most lipid isotopes were resolved chromatographically, we note that CE(22:5) (n3) was not resolved from CE(22:6), and so the signal for this species also contained the M + 2 isomer for CE(22:6). Quantification of lipid classes was determined as the sum composition of the lipid species within each class. For validation of lipid classes, only the 563 lipid species common to both data sets were used for the calculation of lipid classes. Over 90% of the lipid species were measured with a coefficient of variation <20% (based on PQC samples). Batch effects were corrected using a median centring approach utilising PQC samples [96]. Outliers deemed to be of technical origin, such as missed injections (*n* = 19), were excluded from the downstream analysis. Prior to statistical analysis, lipid data were log_10_ transformed. A multivariable linear regression adjusted for sex and BMI was performed to evaluate the associations with age. Similarly, sex-related differences in lipid species were assessed by linear regression adjusting for age and BMI or age, BMI, total cholesterol, HDL-C, and triglycerides. We included clinical lipid measures in the model to be able to identify individual lipid species associated with phenotypes independently of lipoprotein metabolism. Furthermore, we performed age–sex interaction analyses (including an age- and sex-interaction term in the model). For interpretation of results, β-coefficients from linear regression analysis and 95% confidence intervals associated with these were converted to percentage differences (% difference = (10^β-coefficient^ − 1) × 100). To account for false discovery rate, *p*-values were corrected for multiple comparisons using the Benjamini and Hochberg procedure [97]. Sex-stratified heat maps were constructed to visualise lipid classes/species levels across age groups, averaging the lipid levels of participants into 1-year intervals. The averaged lipid class/species levels were normalised to a reference group consisting of the 25- to 34-year–olds. The mean value of each lipid in the reference group was subtracted from all 1-year interval means for that lipid, then the resulting values were scaled by the standard deviation for both sexes in the reference group ([lipid mean in a given age group − reference mean]/reference standard deviation). Lipid ratios (747 × [747 − 1]/2 = 278,631) were generated on the log scale (ratio of lipid a/lipid b is log[a/b] = −log[b/a], so we can refer to ratios as lipid pairs). The association of lipid pairs with phenotypes was assessed using linear models, with the lipid–lipid pair used as the outcome. To evaluate the difference in using lipid ratios compared to individual lipids, the *p*-gain statistic was calculated, defined as the lower phenotype *p*-value from the 2 lipids in the ratio divided by the phenotype *p*-value when using the lipid ratio as the outcome. Here, we applied a conservative cutoff point for the *p*-gain as described previously [28]. All statistical analyses were performed in R (3.5.2).

### Nomenclature of lipids

The naming system for all lipid species reported in this paper follows guidelines developed by LIPID MAPS and others [8, 9]. Some species are reported with the (a), (b), or (c) notations because it is not possible to provide full structural detail for such species. The (a), (b), and (c) denote the elution order from chromatographic column. PE(P-17:0/22:6)(a), e.g., refers to the species that elutes prior to PE(P-17:0/22:6)(b). For most glycerophospholipids that have 2 FA chains, the naming may be based on total FA carbon and double-bond content, e.g., PC(38:6); when the FA chain composition is known whilst the sn1 and sn2 positions are undetermined, PC(38:6) can be expressed as PC(16:0_22:6), or when sn1 and sn2 positions are known, as PC(16:0/22:6). For TG species, we used NL MS scans, and therefore, these species are expressed as total FA composition and the specific NL associated with it; e.g., a TG species composed of 56 FA carbons and 2 double bonds in which the NL corresponds to an 18:2 FA is expressed as TG(56:2) [NL-18:2].

## Supporting information

S1 Fig CV (%). The CVs for each lipid species were computed separately for men (blue circles) and women (pink circles) as follows: (SD/mean concentration) × 100. Each circle represents individual lipid species. See S1 Data for underlying data. CV, coefficient of variation
(TIF)

S2 Fig Pearson’s correlation coefficients between all lipid species in the whole cohort (men and women combined). Pearson’s correlation coefficients were calculated between all pairs of lipid species. The correlation coefficients were plotted as a heat map. The colour scale illustrates the magnitude and direction of correlation between lipid species: red, positive correlations and blue, negative correlations.
(ZIP)

S3 Fig Pearson’s correlation coefficients between all lipid species in men. Pearson’s correlation coefficients were calculated between all pairs of lipid species. The correlation coefficients were plotted as a heat map. The colour scale illustrates the magnitude and direction of correlation between lipid species: red, positive correlations and blue, negative correlations.
(ZIP)

S4 Fig Pearson’s correlation coefficients between all lipid species in women. Pearson’s correlation coefficients were calculated between all pairs of lipid species. The correlation coefficients were plotted as a heat map. The colour scale illustrates the magnitude and direction of correlation between lipid species: red, positive correlations and blue, negative correlations.
(ZIP)

S5 Fig Differences (men relative to women) in the Pearson’s correlation coefficients between all lipid species. The differences in the Pearson’s correlation coefficients were calculated for all pairs (men correlation coefficients subtracted from women’s correlation coefficients). The differences in the correlation coefficients were plotted as a heat map. The colour scale illustrates the magnitude and direction of difference in the correlation of lipid species in men and women.
(ZIP)

S6 Fig Correlation between regression coefficients of lipids associated with sex. The regression coefficients on *x* axis (AusDiab) and *y* axis (Busselton) cohorts have an R^2^ = 0.8398. See S1 Data for underlying data. AusDiab, Australian Diabetes, Obesity and Lifestyle Study.
(TIF)

S7 Fig Associations between age and plasma lipid species. A random-effect meta-analysis between age and log-transformed lipid species concentration was performed on 10,339 individuals (from the AusDiab cohort) and 4,207 participants (from the BHS cohort) adjusting for sex, BMI, and cholesterol, HDL-C, and triglyceride levels. The pooled effect size as percentage difference per year for each lipid species is displayed on the *x* axis. Open circles show nonsignificant species, grey circles show species with *p* < 0.05, and brown circles show the 30 most significant species after correction for multiple comparisons (6.29 × 10^−60^). Whiskers represent 95% confidence intervals. See S1 Data for the underlying data. AusDiab, Australian Diabetes, Obesity and Lifestyle Study; BHS, Busselton Health Study; BMI, body mass index; HDL-C, high-density lipoprotein cholesterol.
(TIF)

S8 Fig Age-related sex differences in plasma lipid class levels in the AusDiab cohort. The cohort was stratified into women (*n* = 5,685, top panel) and men (*n* = 4,654, bottom panel). Average lipid class levels were calculated for each 1-year age interval group and then centred and scaled to a ‘reference’ group (25- to 34-year–old participants). Age groups (by 1-year intervals) are displayed on the *y* axis and the lipid class on the *x* axis. The analysis was adjusted for BMI and clinical lipids. Colour intensities represent the number of standard deviations away from the mean lipid levels of the reference group (25–34 years old). AusDiab, Australian Diabetes, Obesity and Lifestyle Study; BMI, body mass index.
(TIF)

S9 Fig Age-related, sex differences in plasma lipid species levels in the AusDiab cohort. The cohort was stratified into men (*n* = 4,654, left panel) and women (*n* = 5,685, right panel). Average lipid species levels were calculated for each 1-year age interval and then centred and scaled to a ‘reference’ group corresponding to the 25- to 34-year–old participants. Age groups (by 1-year intervals) are displayed on the *x* axis and the lipid species on the *y* axis. Colour intensities represent the number of standard deviations away from the mean lipid levels of the reference group (25–34 years old). AusDiab, Australian Diabetes, Obesity and Lifestyle Study.
(EPS)

S10 Fig Regression coefficients between lipid species and BMI in the AusDiab and Busselton cohorts. The correlation between regression coefficients of each lipid species associated with BMI in the AusDiab (*x* axis) and in the Busselton cohort (*y* axis) was examined. See S1 Data for underlying data. AusDiab, Australian Diabetes, Obesity and Lifestyle Study; BMI, body mass index.
(TIF)

S11 Fig Correlation between metabolic risk factors. Pearson's correlation coefficients were calculated for each pair of risk factors. Colour intensities show the strength of correlation. Significant correlations at *p*-value *p < 0.05, **p < 0.01, and ***p < 0.001, respectively.
(TIF)

S12 Fig Association of BMI, WC, and WHR with lipid species. Linear regression analyses of BMI (A), WC (B), and WHR (C) with lipid species were performed adjusting for age, sex, cholesterol, HDL-C, and triglycerides. Open grey symbols, closed grey symbols, and closed orange symbols show lipid species with corrected *p*-values >0.05, <0.05, and <1 × 10^−11^, respectively. Whiskers represent 95% confidence intervals. See S1 Data for the underlying data. BMI, body mass index; HDL-C, high-density lipoprotein cholesterol; WC, waist circumference; WHR, waist/hip ratio.
(TIF)

S13 Fig Association between BMI, WC, or WHR and the plasma lipidome. A linear regression between log-transformed lipid concentration and BMI or WC or WHR was performed on 10,339 subjects adjusting for age, sex, total cholesterol, HDL-C, and triglycerides. (A) Venn diagram showing overlaps and unique associations of lipid species with BMI, WC, and WHR. (B), (C), and (D) show lipid species significantly associated with BMI only, WHR, only, and WC only, respectively. See S1 Data for the underlying data. BMI, body mass index; HDL-C, high-density lipoprotein cholesterol; WC, waist circumference; WHR, waist/hip ratio.
(TIF)

S14 Fig Association of smoking with plasma lipidomic profile. A logistic regression analysis between smoking status and log_10_-transformed lipid species concentrations was performed adjusting for age, sex, BMI, total cholesterol, HDL-C, and triglycerides. Grey circles show nonsignificant species (*p* > 0.05), and grey and pink circles show species with *p* < 0.05 and *p* < 2.95 × 10^−9^, respectively, after correction for multiple comparisons. The whiskers represent 95% confidence intervals. See S1 Data for underlying data. BMI, body mass index; HDL-C, high-density lipoprotein cholesterol.
(TIF)

S15 Fig Correlation matrix between fatty acid composition of lysophospholipids. Pearson's correlation analysis was performed between 27 fatty acids. Blue coloured eclipses in each square represent positive correlations, and orange show negative correlations. See S1 Data for the underlying data.
(TIF)

S16 Fig Association between age, sex, and BMI with plasma lysophosphatidylcholine fatty acids. Multivariable linear regression analysis of age (A), sex (B), and BMI (C) with log-transformed lipid composition data was performed on 10,339 AusDiab participants adjusting for BMI, age, and sex (as appropriate) together with total cholesterol, HDL-C, and triglycerides. Open grey circles represent nonsignificant fatty acids (corrected *p* > 0.05). Orange circles show fatty acids associated with BMI, age, or sex (corrected *p* < 0.05). Bars represent the 95% confidence intervals. See S1 Data for the underlying data. AusDiab, Australian Diabetes, Obesity and Lifestyle Study; BMI, body mass index; HDL-C, high-density lipoprotein cholesterol.
(TIF)

S17 Fig Selected lipid ratios and sphingolipid metabolic pathway associated with BMI. A linear regression adjusted for age, sex, total cholesterol, HDL-C, and triglycerides was performed between individual lipids or lipid concentration ratios and BMI. Each of the panels from (A)–(F) represent association of BMI with a given lipid ratio and individual lipids species that make up the ratio. (G) An overview of the sphingolipid biosynthetic pathway. (H) FADS3 as a sphingoid base desaturase responsible for increased d18:2/d18:1 sphingolipid ratio. BMI, body mass index; DEGS, dihydroceramide desaturase; FADS3, fatty acid desaturase 3; GCS, glucosylceramide synthase; HDL-C, high-density lipoprotein cholesterol; SMase, sphingomyelinase; SMS, sphingomyelin synthase.
(TIF)

S18 Fig The d18:2 to d18:1 ratios of sphingolipid species associated with sex. A linear regression adjusted for age, BMI, total cholesterol, HDL-C, and triglycerides was performed between individual lipids or lipid concentration ratios and sex. (A) represents association of sex with the ratio between d18:2/d18:1 sphingolipid. (B) FADS3 as a sphingoid base desaturase responsible for the conversion of d18:2/d18:1 sphingolipid. BMI, body mass index; FADS3, fatty acid desaturase 3; HDL-C, high-density lipoprotein cholesterol.
(TIF)

S19 Fig Association of BMI with the ratio between PE(P)/PE(O). A linear regression adjusted for age, sex, total cholesterol, HDL-C, and triglycerides was performed between BMI and individual lipid or lipid concentration ratio. BMI, body mass index; HDL-C, high-density lipoprotein cholesterol; PE(O), alkylphosphatidylethanolamine; PE(P), alkenylphosphatidylethanolamine.
(TIF)

S1 Table The correlation structure between all lipid species in the whole cohort (men and women combined). (XLSX)

S2 Table The correlation structure between all lipid species in men. (XLSX)

S3 Table The correlation structure between all lipid species in women. (XLSX)

S4 Table Differences in the Pearson's correlations (men relative to women) for all lipid species. (XLSX)

S5 Table Validation of the association between sex and lipid species. (XLSX)

S6 Table Characteristics of women participants. (XLSX)

S7 Table Validation of the association of BMI with lipid species. BMI, body mass index.
(XLSX)

S8 Table Association of BMI with lipid concentration ratios. BMI, body mass index.
(XLSX)

S9 Table Association of plasma phospholipid fatty acid composition with BMI, age, or sex. BMI, body mass index.
(XLSX)

S10 Table Association of age with lipid concentration ratios. (XLSX)

S11 Table Association of sex with lipid concentration ratios. (CSV)

S12 Table Association of sphingolipid ratios with BMI, age, or sex. BMI, body mass index.
(XLSX)

S13 Table Association of the d182:1/d181 sphingoid base ratios with BMI, age, or sex. BMI, body mass index.
(XLSX)

S14 Table Association of the PE(P)/PE(O) ratios with BMI, age, or sex. BMI, body mass index; PE(O), alkylphosphatidylethanolamine; PE(P), alkenylphosphatidylethanolamine.
(XLSX)

S15 Table SwissLipids identifiers for lipid species. (XLSX)

S16 Table Characteristics of study participants. (XLSX)

S17 Table MRM transitions and conditions for examined lipid species. MRM, multiple reaction monitoring.
(XLSX)

S1 Data Numerical data underlying figures and supplemental figures. (XLSX)

## Abbreviations

ApoB: apolipoprotein B
AusDiab: Australian Diabetes, Obesity and Lifestyle Study
BHS: Busselton Health Study
BMI: body mass index
CE: cholesteryl ester
Cer: ceramide
Cer-1-P: ceramide-1-phosphate
CV: coefficient of variation
CVD: cardiovascular disease
DE: dehydrocholesterol
deoxyCer: deoxyceramide
DG: diacylglycerol
DHA: docosahexaenoic acid
dhCer: dihydroceramide
DPA: docosapentaenoic acid
ELOVL: elongation of very long chain fatty acids protein
EPA: eicosapentaenoic acid
FADS3: fatty acid desaturase 3
FDR: false discovery rate
GM1: G_M1_ ganglioside
GM3: G_M3_ ganglioside
HDL-C: high-density lipoprotein cholesterol
HexCer: monohexosylceramide
Hex2Cer: dihexosylceramide
Hex3Cer: trihexosylceramide
HRT: hormone replacement treatment
LCAT: lecithin cholesterol acyltransferase
LC-ESI-MS/MS: LC electrospray ionisation MS/MS
LC-MS/MS: liquid chromatography tandem mass-spectrometry
LDL-C: low-density lipoprotein cholesterol
LPC: lysophosphatidylcholine
LPC(O): lysoalkylphosphatidylcholine
LPC(P): lysoalkenylphosphatidylcholine
LPE: lysophosphatidylethanolamine
LPE(P): lysoalkenylphosphatidylethanolamine
LPI: lysophosphatidylinositol
NIST: National Institute of Standards and Technology
NL: neutral loss
PC: phosphatidylcholine
PC(O): alkylphosphatidylcholine
PC(P): alkenylphosphatidylcholine
PE: phosphatidylethanolamine
PE(O): alkylphosphatidylethanolamine
PE(P): alkenylphosphatidylethanolamine
PG: phosphatidylglycerol
PI: phosphatidylinositol
PLA2: phospholipase A2
PQC: plasma quality control
PS: phosphatidylserine
SCD-1: stearoyl CoA desaturase 1
SM: sphingomyelin
sn: stereospecifically numbered
Sph: sphingosine
SPT: serine palmitoyltransferase
S-1-P: sphingosine-1-phosphate
TG: triacylglycerol
TG(O): alkyl-diacylglycerol
TQC: technical quality control
T2D: type 2 diabetes
UWA HREC: University of Western Australia Human Research Ethics Committee
VLDL-C: very low-density lipoprotein cholesterol
WC: waist circumference
WHR: waist/hip ratio
