# Correction: High-coverage plasma lipidomics reveals novel sex-specific lipidomic fingerprints of age and BMI: Evidence from two large population cohort studies

**DOI:** 10.1371/journal.pbio.3001049

**Published:** 2020-12-09

**Authors:** Habtamu B. Beyene, Gavriel Olshansky, Adam Alexander T. Smith, Corey Giles, Kevin Huynh, Michelle Cinel, Natalie A. Mellett, Gemma Cadby, Joseph Hung, Jennie Hui, John Beilby, Gerald F. Watts, Jonathan E. Shaw, Eric K. Moses, Dianna J. Magliano, Peter J. Meikle

The thirteenth author's name is spelled incorrectly. The correct name is: Jonathan E. Shaw

## References

[1] Beyene HB, Olshansky G, T. Smith AA, Giles C, Huynh K, Cinel M, et al (2020) High-coverage plasma lipidomics reveals novel sex-specific lipidomic fingerprints of age and BMI: Evidence from two large population cohort studies. PLoS Biol 18(9): e3000870 10.1371/journal.pbio.3000870 32986697 PMC7544135

