# Supplementary figures and images for: High-coverage plasma lipidomics reveals novel sex-specific lipidomic fingerprints of age and BMI: Evidence from two large population cohort studies
Source: PLoS Biol. 2020 Sep 28;18(9):e3000870. doi: 10.1371/journal.pbio.3000870 (PMC7544135; doi:10.1371/journal.pbio.3000870)

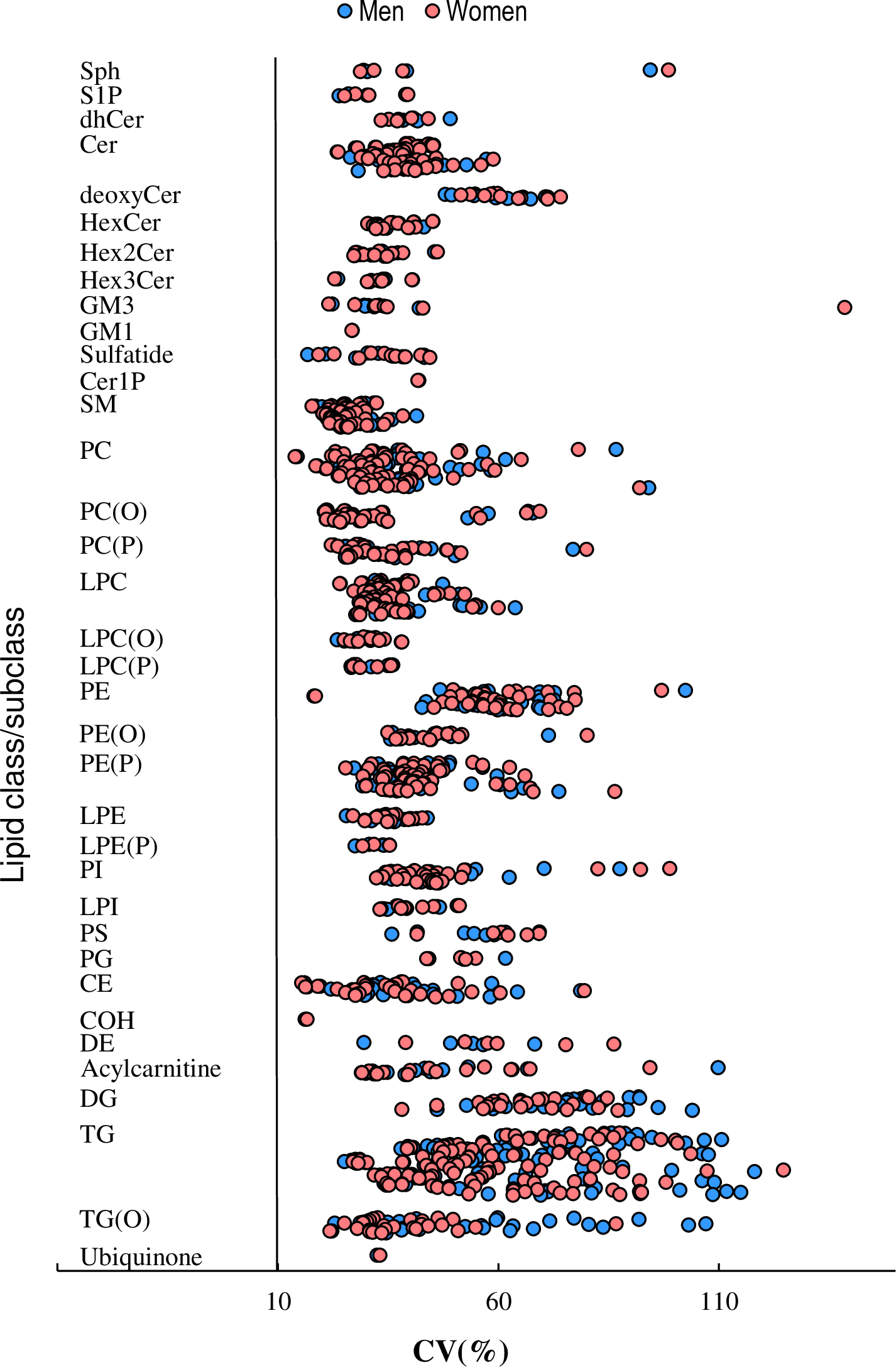

Supplement: S1 Fig — The CVs for each lipid species were computed separately for men (blue circles) and women (pink circles) as follows: (SD/mean concentration) × 100. Each circle represents individual lipid species. See S1 Data for underlying data. CV, coefficient of variation (TIF) [file pbio.3000870.s001.tif]

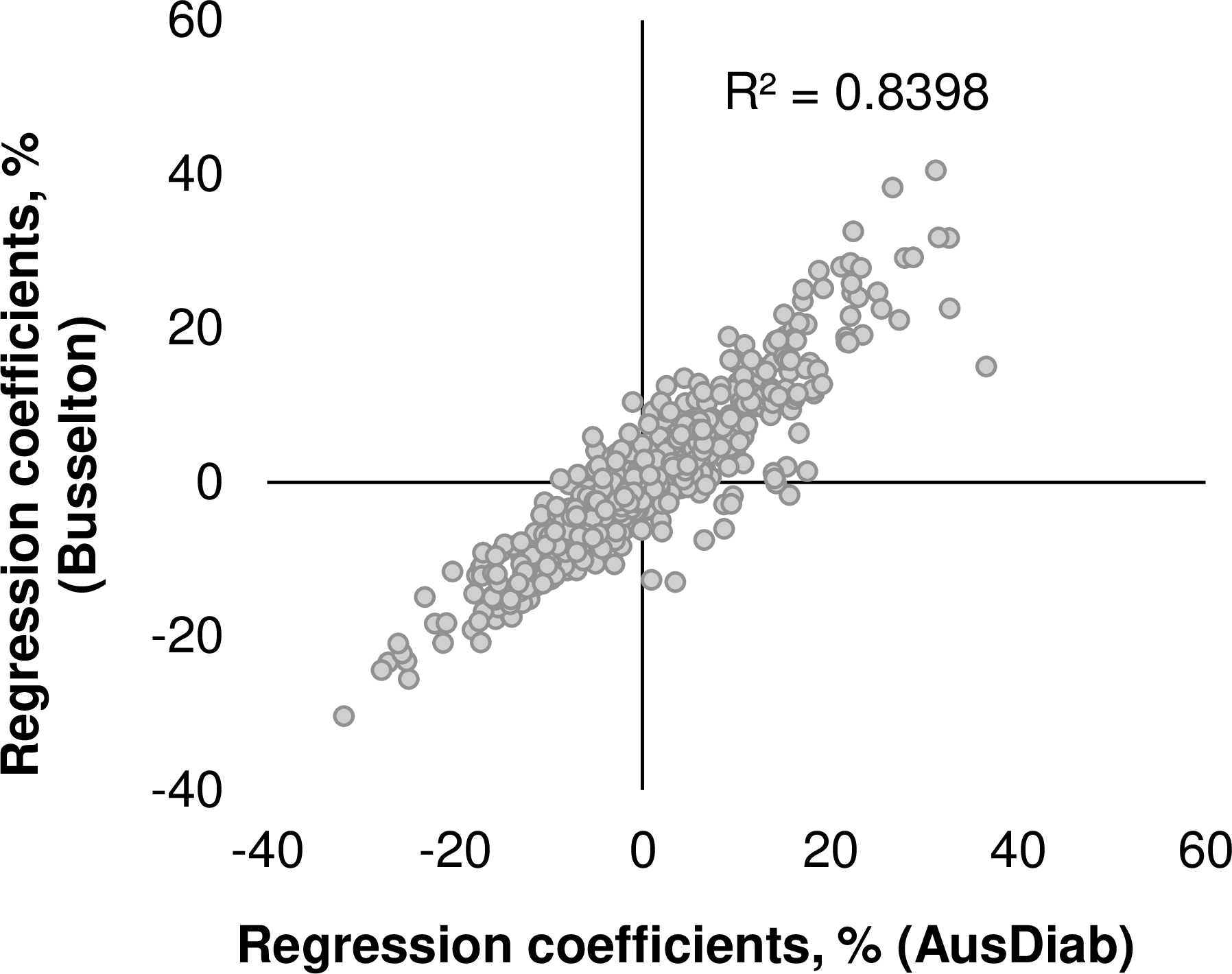

Supplement: S6 Fig — The regression coefficients on x axis (AusDiab) and y axis (Busselton) cohorts have an R2 = 0.8398. See S1 Data for underlying data. AusDiab, Australian Diabetes, Obesity and Lifestyle Study. (TIF) [file pbio.3000870.s006.tif]

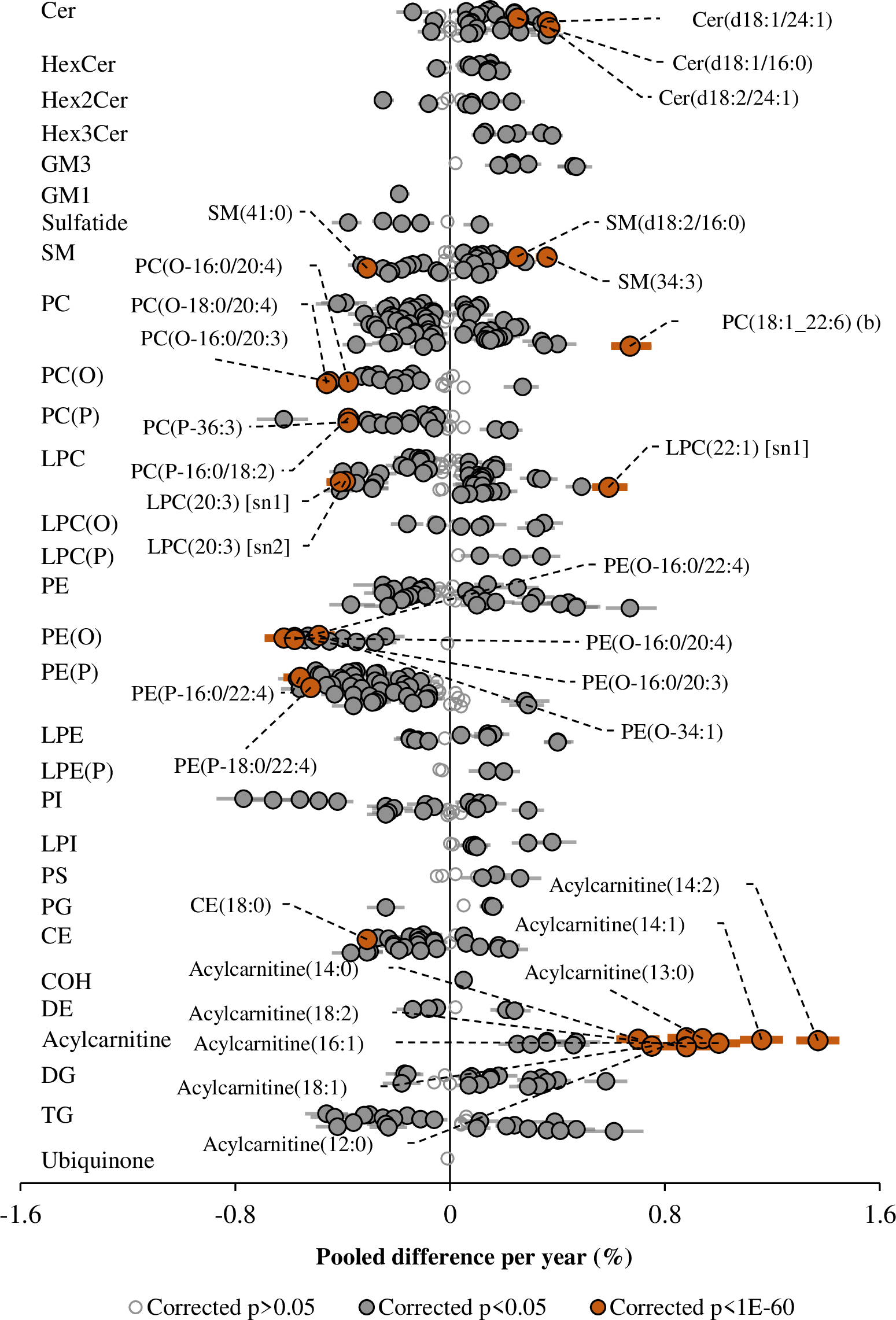

Supplement: S7 Fig — A random-effect meta-analysis between age and log-transformed lipid species concentration was performed on 10,339 individuals (from the AusDiab cohort) and 4,207 participants (from the BHS cohort) adjusting for sex, BMI, and cholesterol, HDL-C, and triglyceride levels. The pooled effect size as percentage difference per year for each lipid species is displayed on the x axis. Open circles show nonsignificant species, grey circles show species with p < 0.05, and brown circles show the 30 most significant species after correction for multiple comparisons (6.29 × 10^−60^). Whiskers represent 95% confidence intervals. See S1 Data for the underlying data. AusDiab, Australian Diabetes, Obesity and Lifestyle Study; BHS, Busselton Health Study; BMI, body mass index; HDL-C, high-density lipoprotein cholesterol. (TIF) [file pbio.3000870.s007.tif]

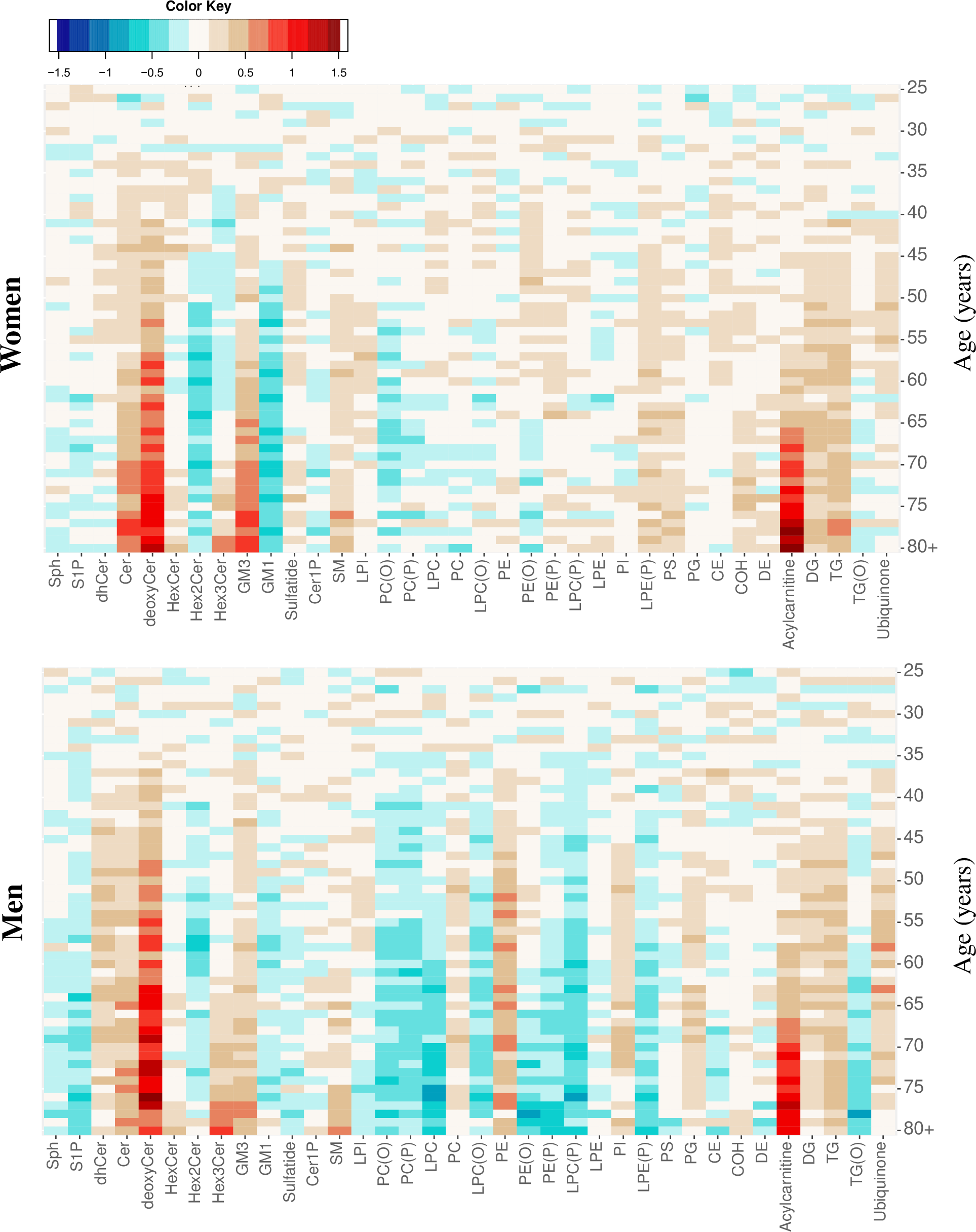

Supplement: S8 Fig — The cohort was stratified into women (n = 5,685, top panel) and men (n = 4,654, bottom panel). Average lipid class levels were calculated for each 1-year age interval group and then centred and scaled to a ‘reference’ group (25- to 34-year–old participants). Age groups (by 1-year intervals) are displayed on the y axis and the lipid class on the x axis. The analysis was adjusted for BMI and clinical lipids. Colour intensities represent the number of standard deviations away from the mean lipid levels of the reference group (25–34 years old). AusDiab, Australian Diabetes, Obesity and Lifestyle Study; BMI, body mass index. (TIF) [file pbio.3000870.s008.tif]

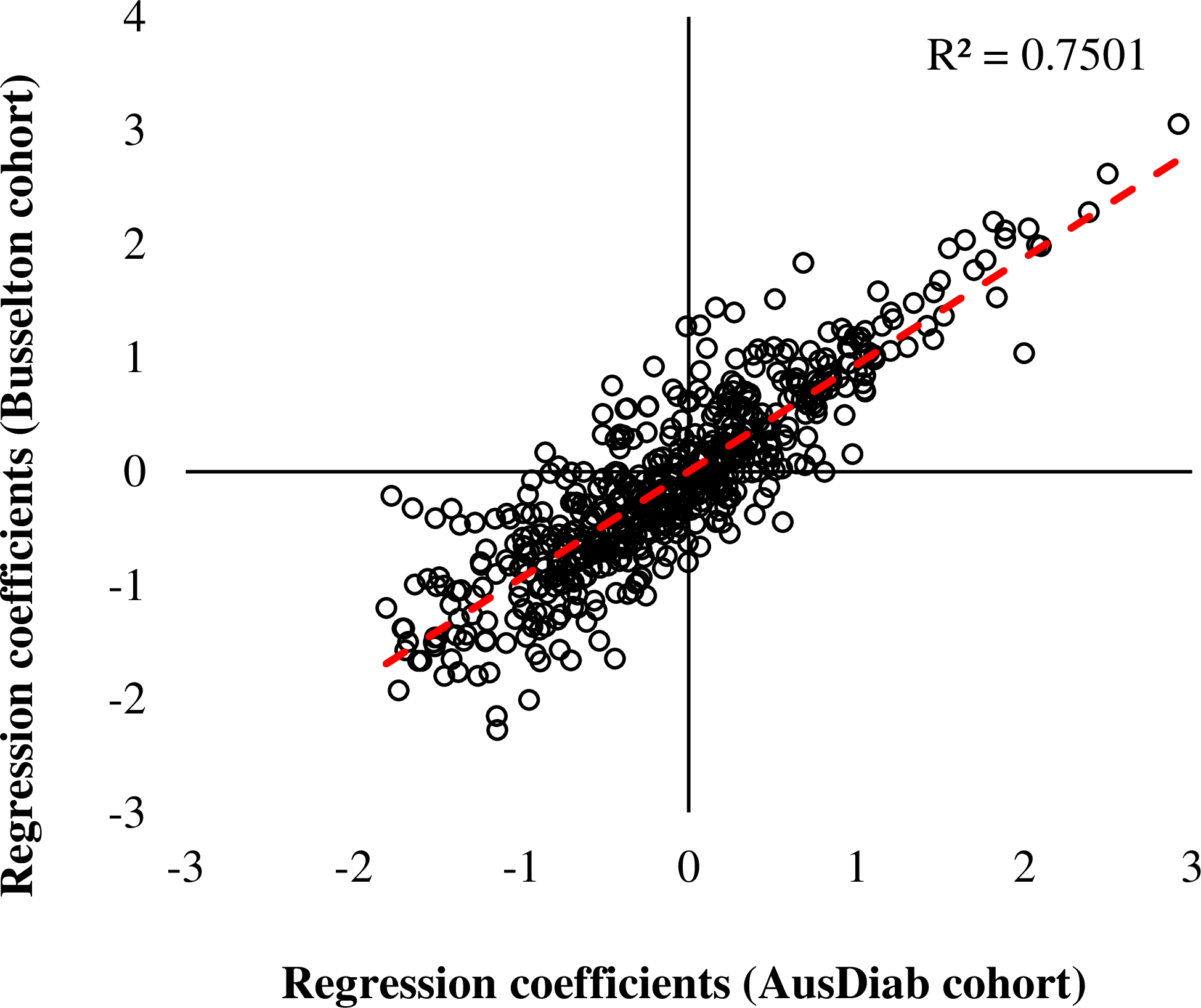

Supplement: S10 Fig — The correlation between regression coefficients of each lipid species associated with BMI in the AusDiab (x axis) and in the Busselton cohort (y axis) was examined. See S1 Data for underlying data. AusDiab, Australian Diabetes, Obesity and Lifestyle Study; BMI, body mass index. (TIF) [file pbio.3000870.s010.tif]

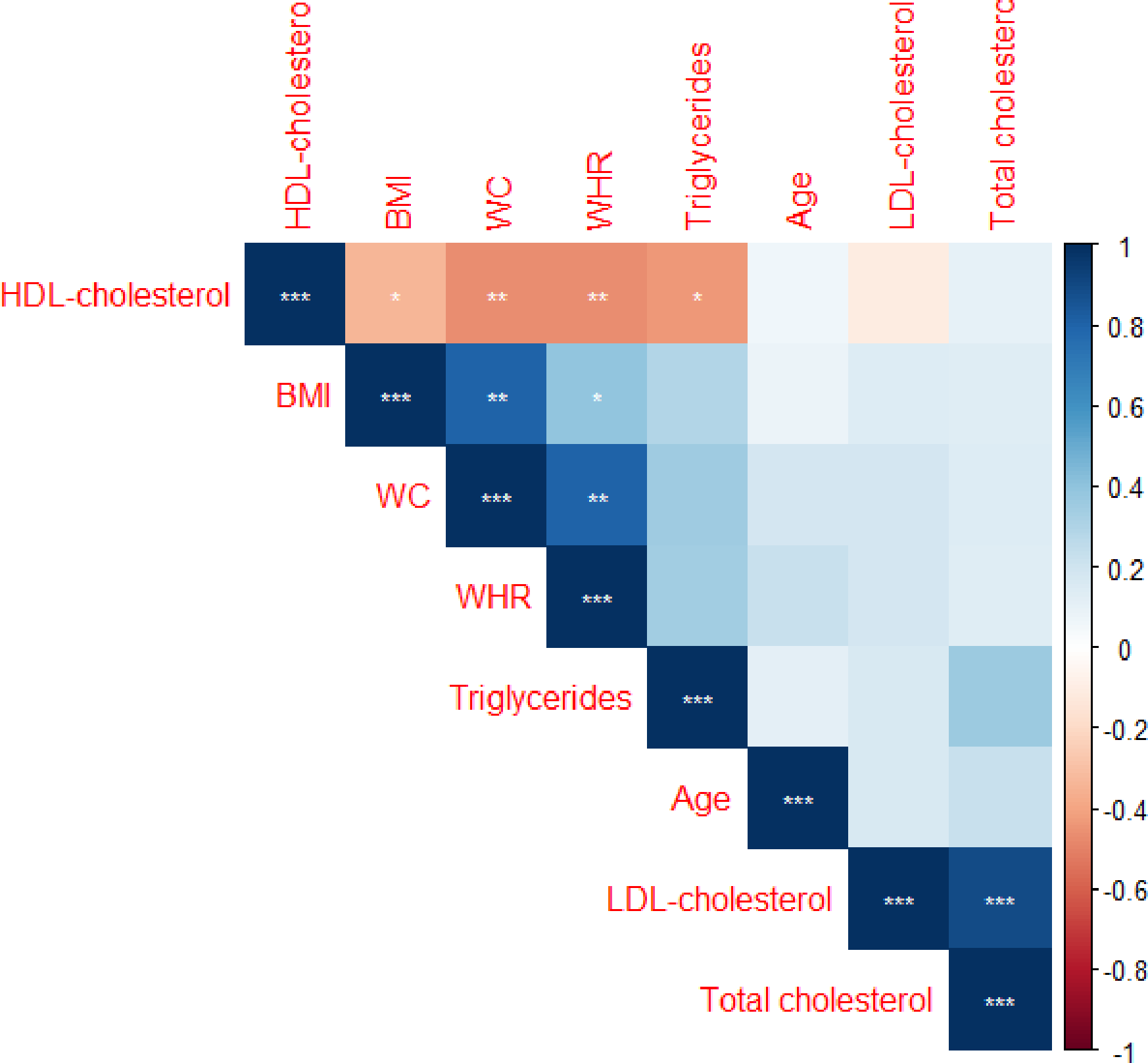

Supplement: S11 Fig — Pearson's correlation coefficients were calculated for each pair of risk factors. Colour intensities show the strength of correlation. Significant correlations at p-value *p < 0.05, **p < 0.01, and ***p < 0.001, respectively. (TIF) [file pbio.3000870.s011.tif]

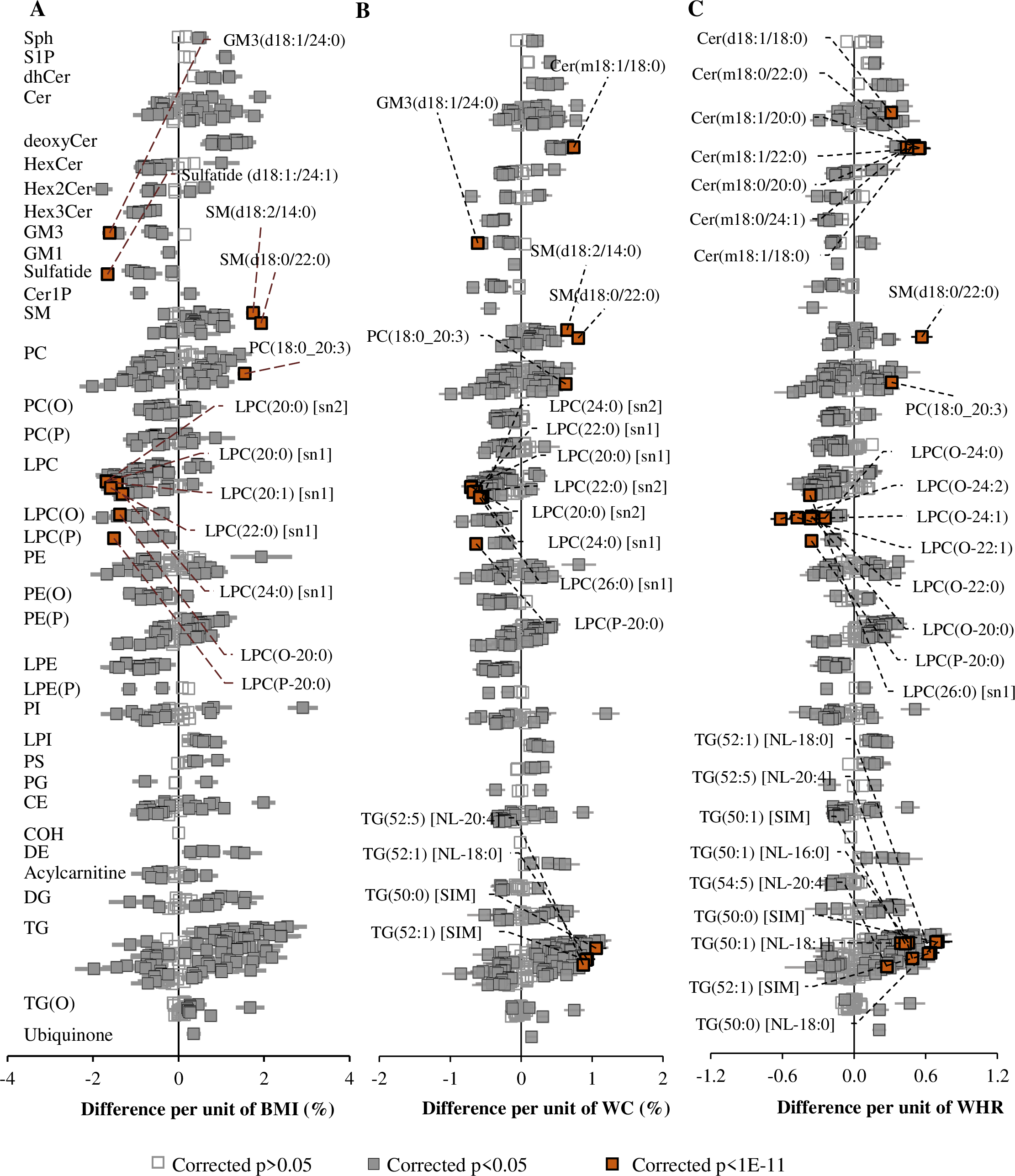

Supplement: S12 Fig — Linear regression analyses of BMI (A), WC (B), and WHR (C) with lipid species were performed adjusting for age, sex, cholesterol, HDL-C, and triglycerides. Open grey symbols, closed grey symbols, and closed orange symbols show lipid species with corrected p-values >0.05, <0.05, and <1 × 10^−11^, respectively. Whiskers represent 95% confidence intervals. See S1 Data for the underlying data. BMI, body mass index; HDL-C, high-density lipoprotein cholesterol; WC, waist circumference; WHR, waist/hip ratio. (TIF) [file pbio.3000870.s012.tif]

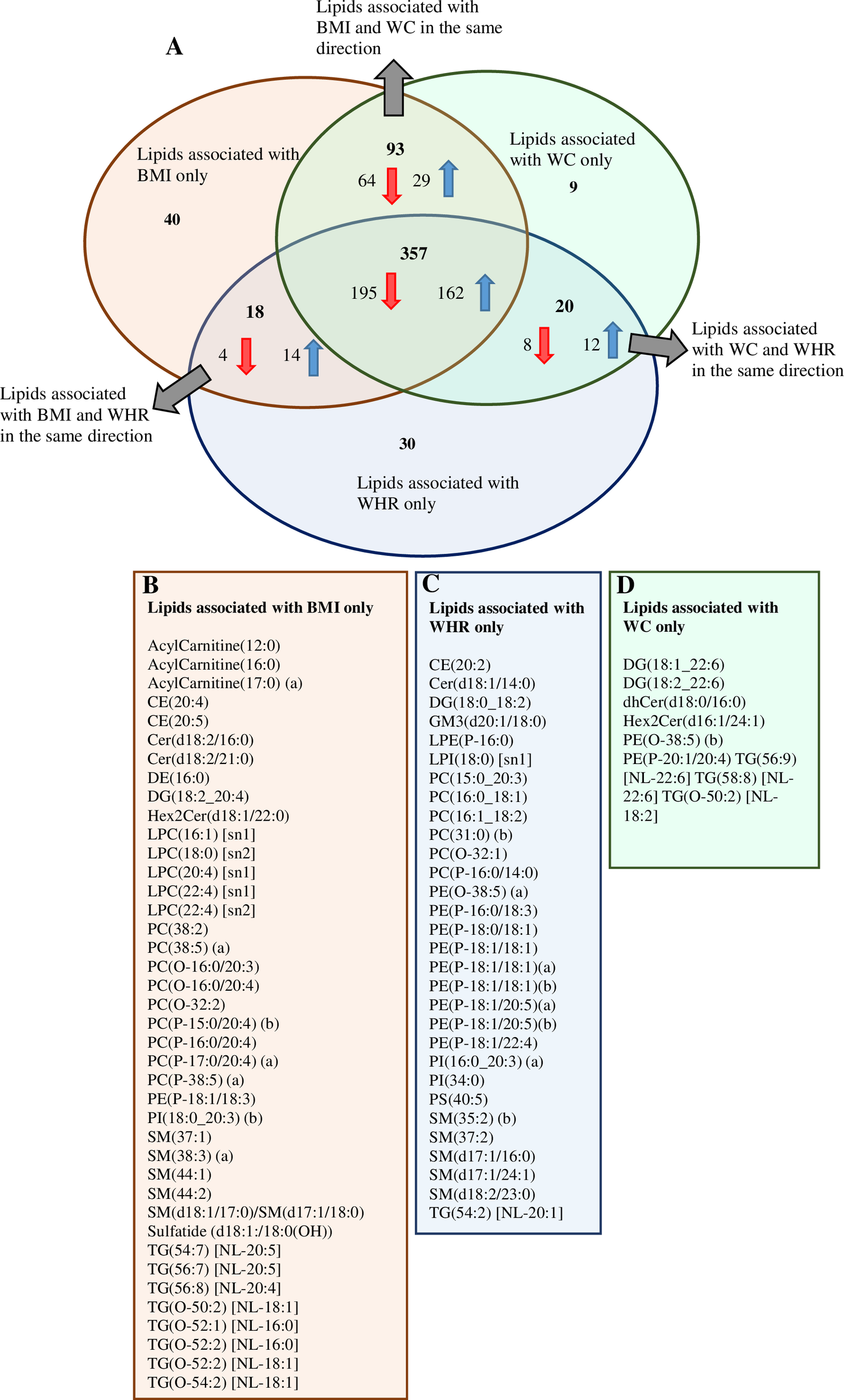

Supplement: S13 Fig — A linear regression between log-transformed lipid concentration and BMI or WC or WHR was performed on 10,339 subjects adjusting for age, sex, total cholesterol, HDL-C, and triglycerides. (A) Venn diagram showing overlaps and unique associations of lipid species with BMI, WC, and WHR. (B), (C), and (D) show lipid species significantly associated with BMI only, WHR, only, and WC only, respectively. See S1 Data for the underlying data. BMI, body mass index; HDL-C, high-density lipoprotein cholesterol; WC, waist circumference; WHR, waist/hip ratio. (TIF) [file pbio.3000870.s013.tif]

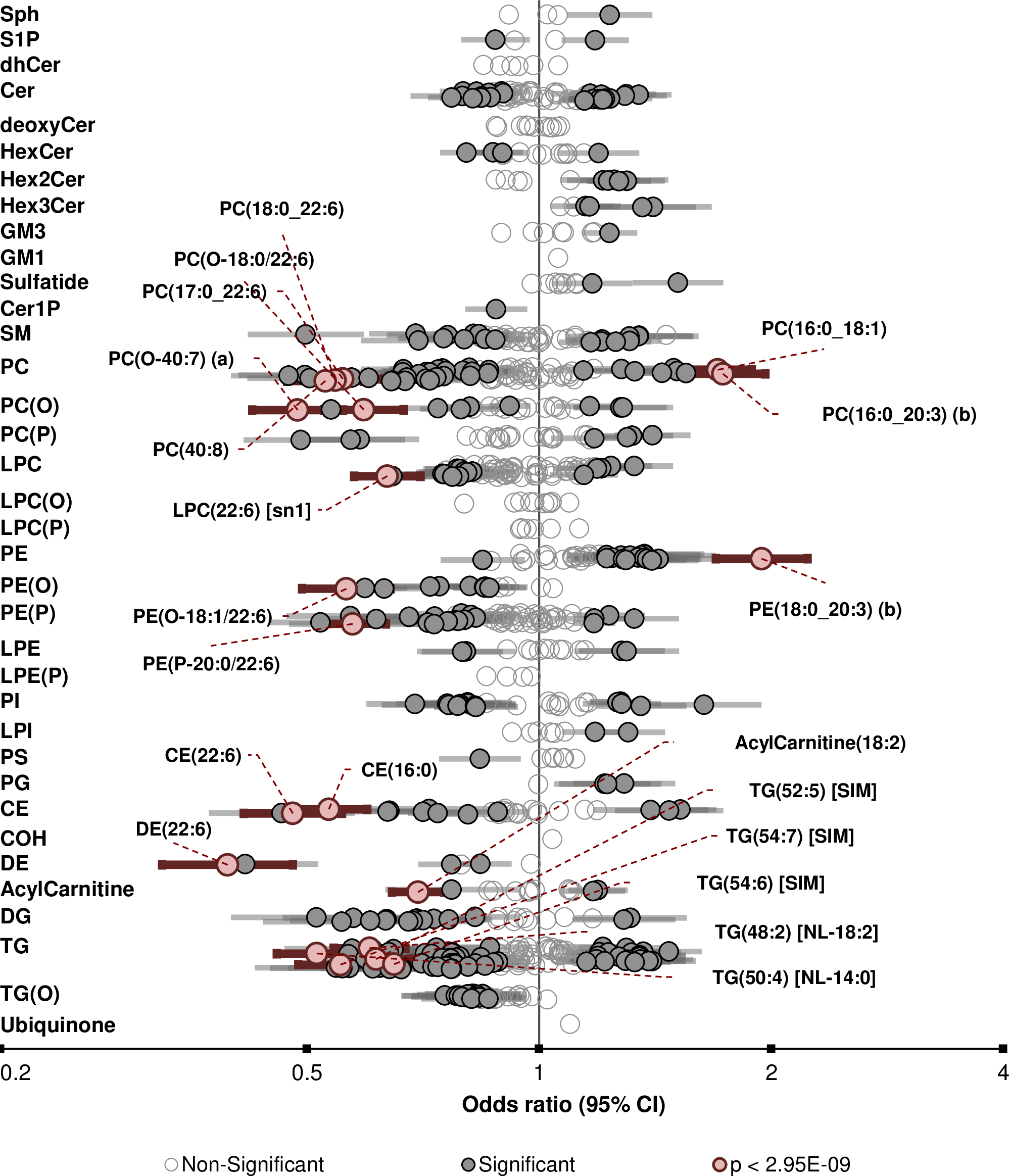

Supplement: S14 Fig — A logistic regression analysis between smoking status and log10-transformed lipid species concentrations was performed adjusting for age, sex, BMI, total cholesterol, HDL-C, and triglycerides. Grey circles show nonsignificant species (p > 0.05), and grey and pink circles show species with p < 0.05 and p < 2.95 × 10^−9^, respectively, after correction for multiple comparisons. The whiskers represent 95% confidence intervals. See S1 Data for underlying data. BMI, body mass index; HDL-C, high-density lipoprotein cholesterol. (TIF) [file pbio.3000870.s014.tif]

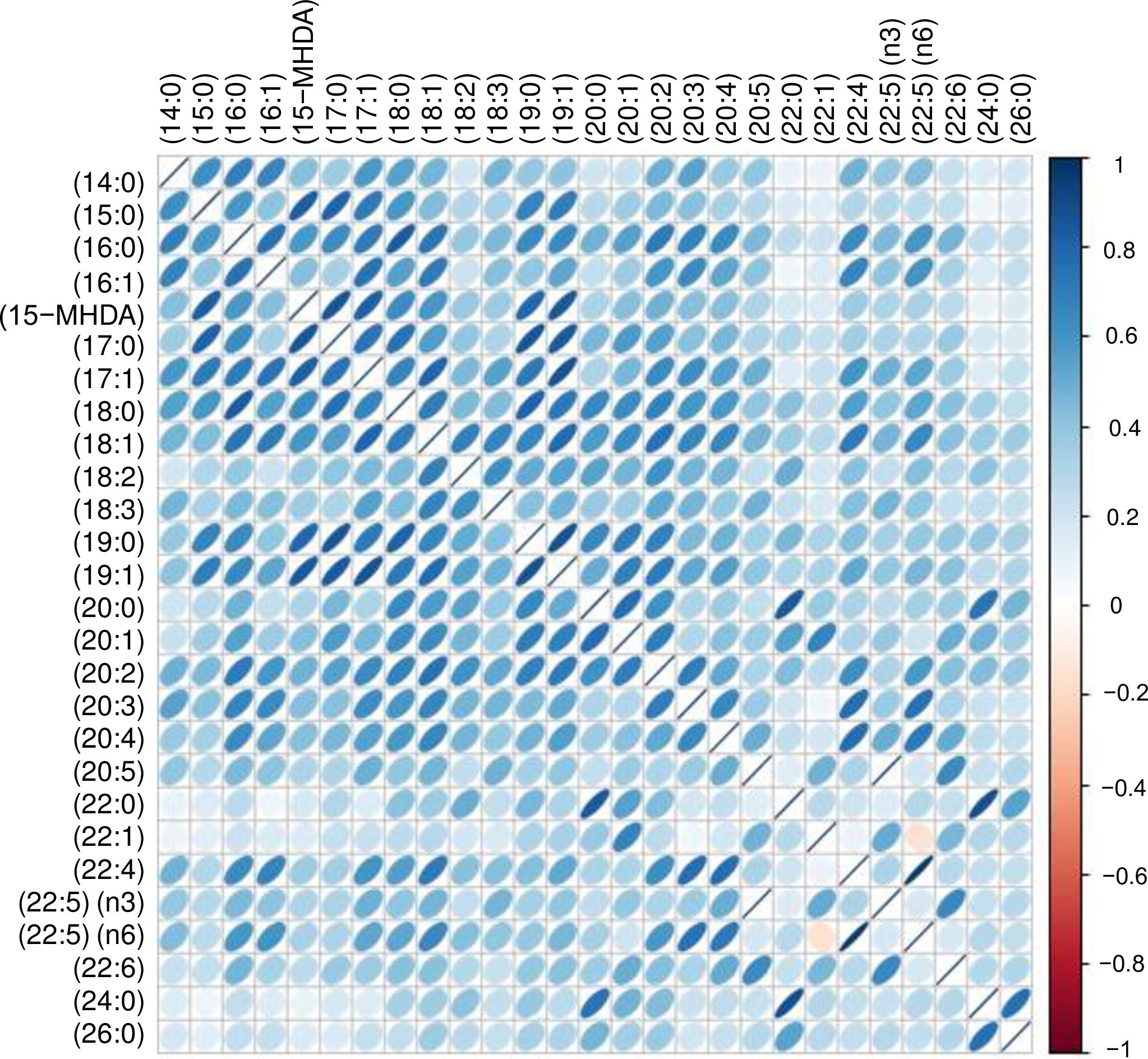

Supplement: S15 Fig — Pearson's correlation analysis was performed between 27 fatty acids. Blue coloured eclipses in each square represent positive correlations, and orange show negative correlations. See S1 Data for the underlying data. (TIF) [file pbio.3000870.s015.tif]

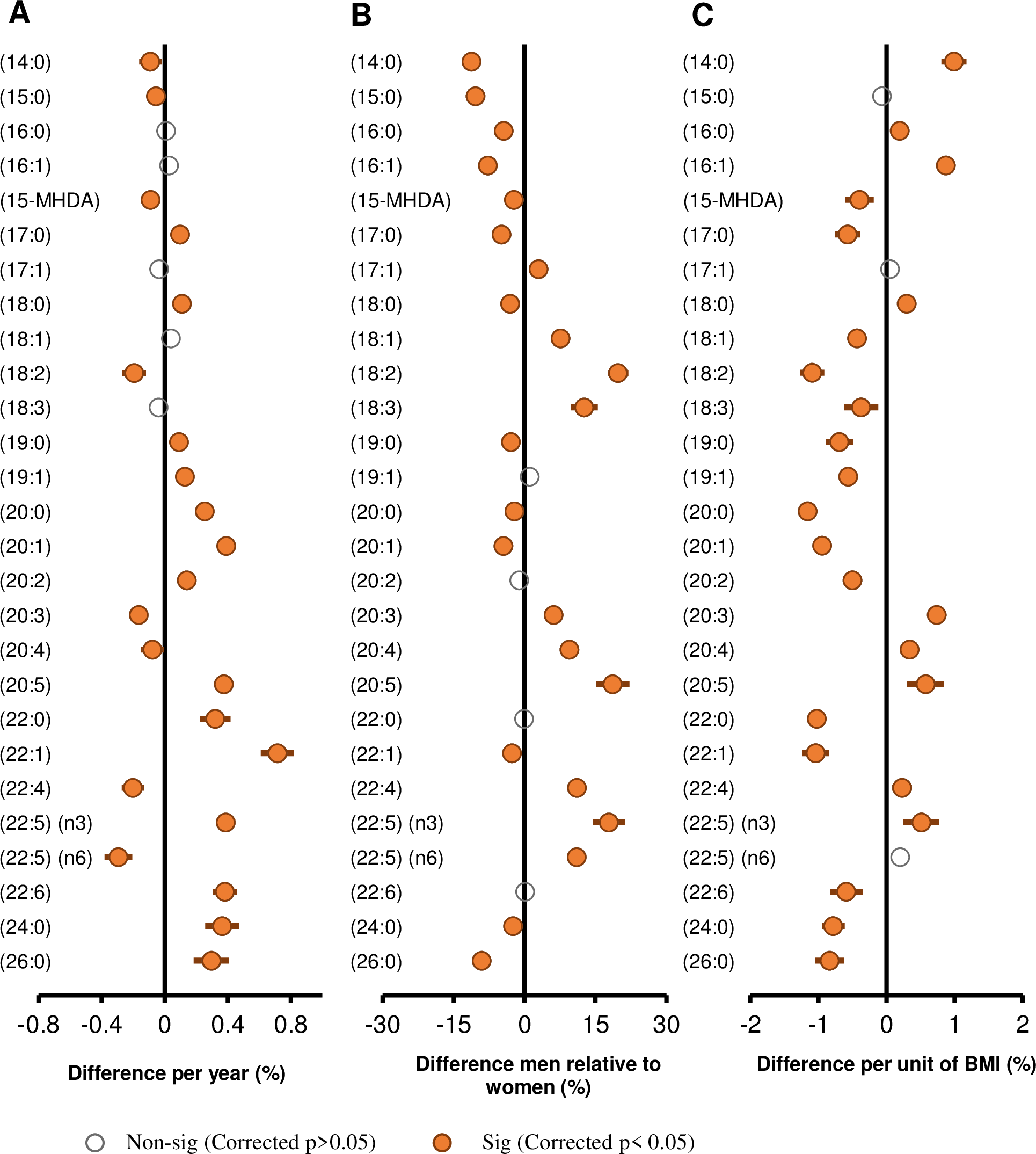

Supplement: S16 Fig — Multivariable linear regression analysis of age (A), sex (B), and BMI (C) with log-transformed lipid composition data was performed on 10,339 AusDiab participants adjusting for BMI, age, and sex (as appropriate) together with total cholesterol, HDL-C, and triglycerides. Open grey circles represent nonsignificant fatty acids (corrected p > 0.05). Orange circles show fatty acids associated with BMI, age, or sex (corrected p < 0.05). Bars represent the 95% confidence intervals. See S1 Data for the underlying data. AusDiab, Australian Diabetes, Obesity and Lifestyle Study; BMI, body mass index; HDL-C, high-density lipoprotein cholesterol. (TIF) [file pbio.3000870.s016.tif]

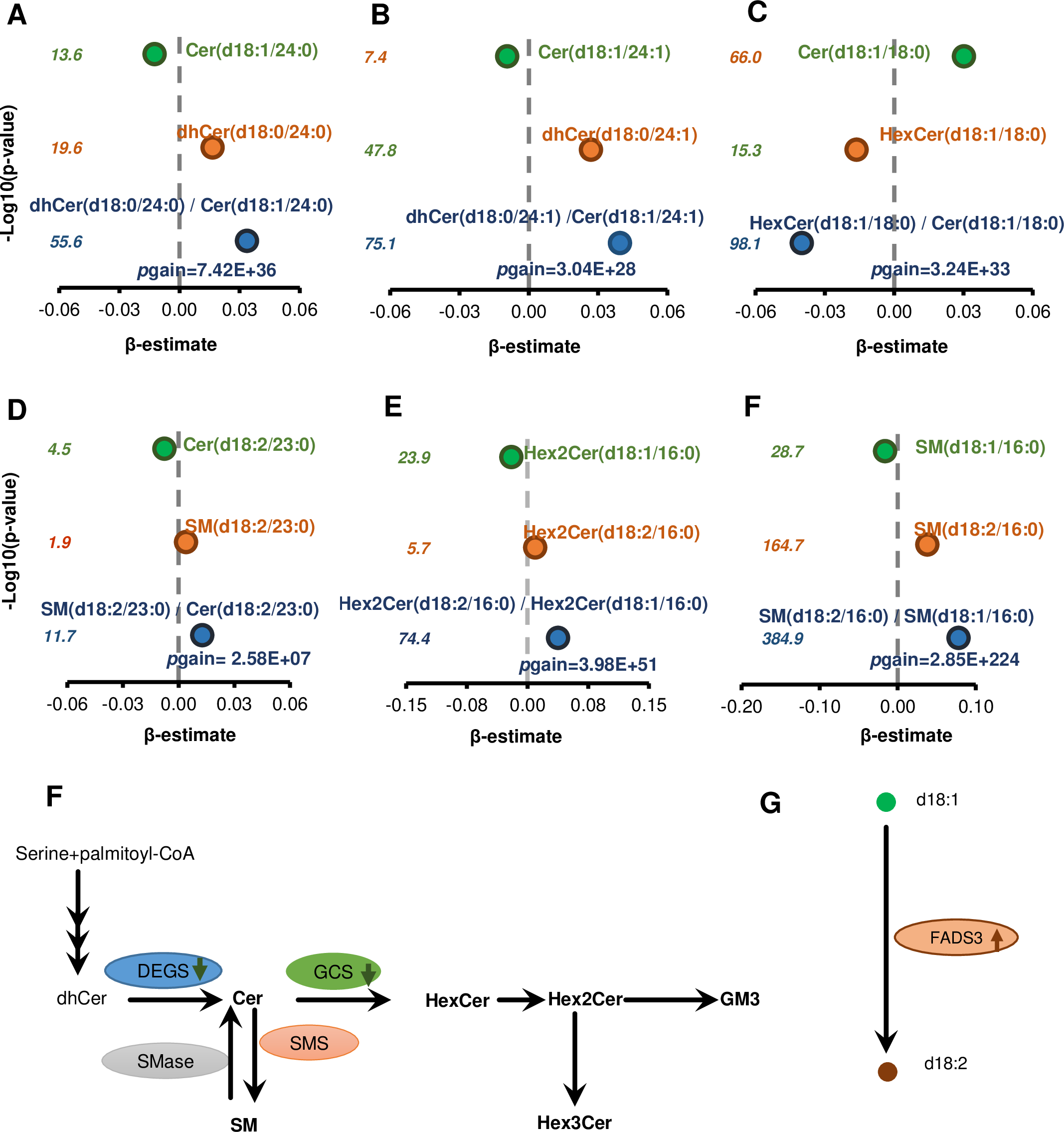

Supplement: S17 Fig — A linear regression adjusted for age, sex, total cholesterol, HDL-C, and triglycerides was performed between individual lipids or lipid concentration ratios and BMI. Each of the panels from (A)–(F) represent association of BMI with a given lipid ratio and individual lipids species that make up the ratio. (G) An overview of the sphingolipid biosynthetic pathway. (H) FADS3 as a sphingoid base desaturase responsible for increased d18:2/d18:1 sphingolipid ratio. BMI, body mass index; DEGS, dihydroceramide desaturase; FADS3, fatty acid desaturase 3; GCS, glucosylceramide synthase; HDL-C, high-density lipoprotein cholesterol; SMase, sphingomyelinase; SMS, sphingomyelin synthase. (TIF) [file pbio.3000870.s017.tif]

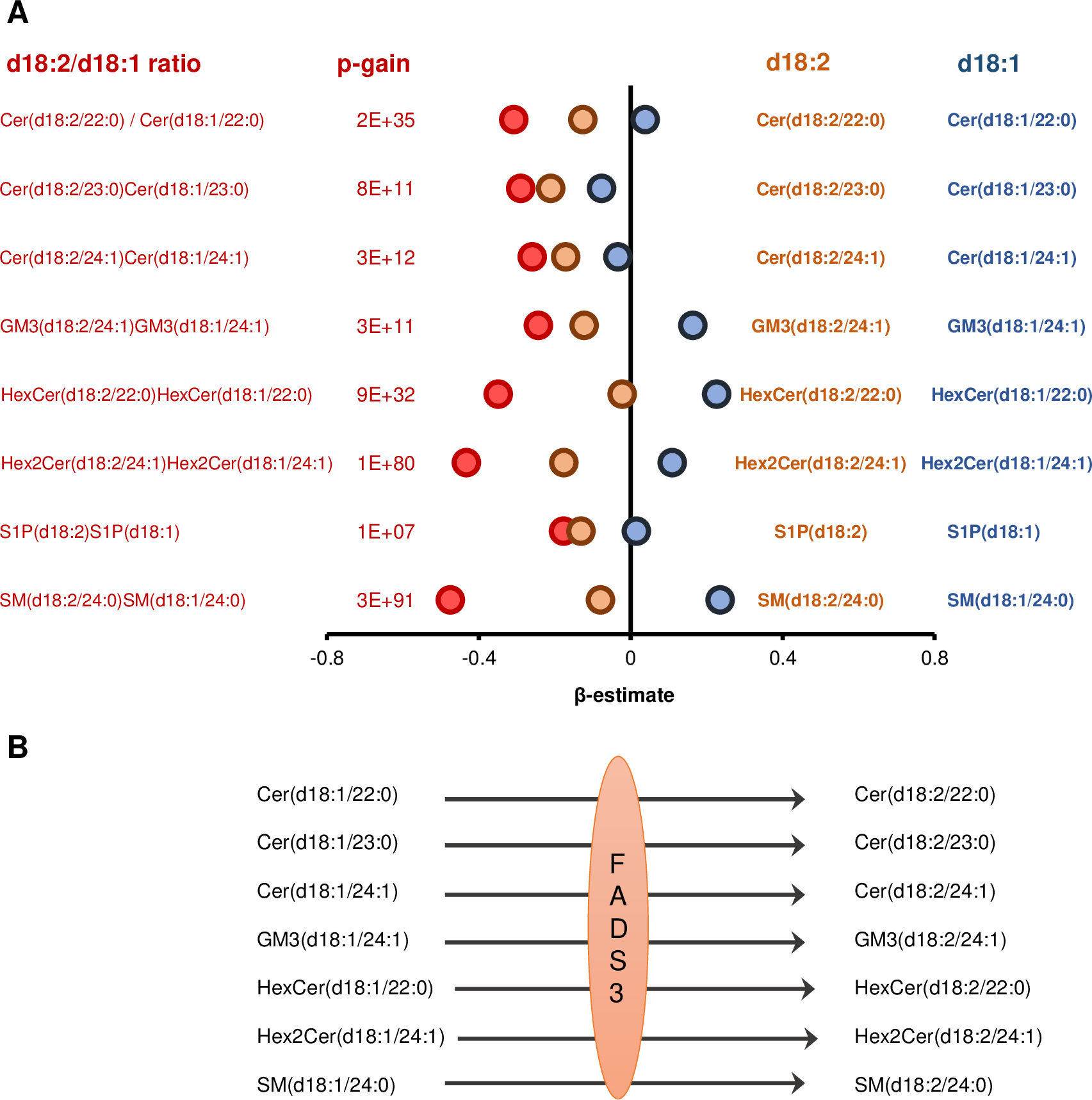

Supplement: S18 Fig — A linear regression adjusted for age, BMI, total cholesterol, HDL-C, and triglycerides was performed between individual lipids or lipid concentration ratios and sex. (A) represents association of sex with the ratio between d18:2/d18:1 sphingolipid. (B) FADS3 as a sphingoid base desaturase responsible for the conversion of d18:2/d18:1 sphingolipid. BMI, body mass index; FADS3, fatty acid desaturase 3; HDL-C, high-density lipoprotein cholesterol. (TIF) [file pbio.3000870.s018.tif]

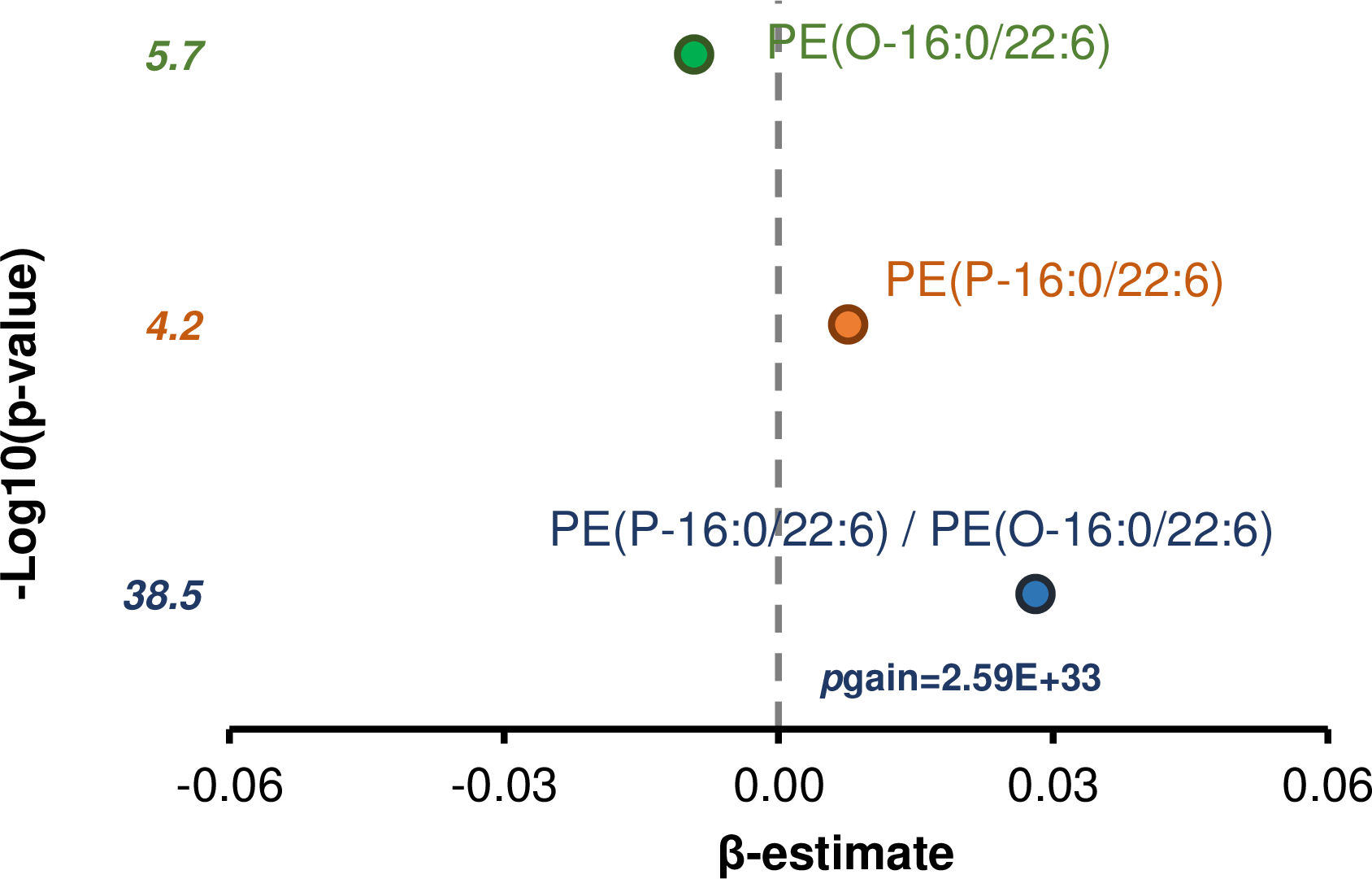

Supplement: S19 Fig — A linear regression adjusted for age, sex, total cholesterol, HDL-C, and triglycerides was performed between BMI and individual lipid or lipid concentration ratio. BMI, body mass index; HDL-C, high-density lipoprotein cholesterol; PE(O), alkylphosphatidylethanolamine; PE(P), alkenylphosphatidylethanolamine. (TIF) [file pbio.3000870.s019.tif]
